# Microstructural disease and hypoperfusion in dilated cardiomyopathy underpin midwall septal fibrosis^☆^

**DOI:** 10.1016/j.jocmr.2026.102749

**Published:** 2026-05-29

**Authors:** Fiona TS Chan, Sam Coveney, Sean L. Zheng, Matthew Webber, George Joy, Hunain Shiwani, Constantin-Cristian Topriceanu, Debbie Falconer, Emma Martin, Matthew Stanley, Iain Pierce, Irvin Teh, Jurgen Schneider, Christopher Nguyen, Alun D. Hughes, James C. Moon, Pier D. Lambiase, Peter Kellman, Erica Dall’Armellina, Gabriella Captur

**Affiliations:** a UCL Unit for Lifelong Health and Ageing, University College London, London, United Kingdom; b UCL Institute of Cardiovascular Science, University College London, London, United Kingdom; c Royal Free Hospital, Centre for Inherited Cardiac Conditions, Cardiology Department, Pond Street, Hampstead, London, United Kingdom; d Biomedical Imaging Science Department, Leeds Institute of Cardiovascular and Metabolic Medicine, University of Leeds, Leeds, United Kingdom; e National Heart and Lung Institute, Imperial College London, London, United Kingdom; f Laboratory of Medical Sciences, Medical Research Council, London, United Kingdom; g Barts Heart Centre, the Cardiovascular Magnetic Resonance Unit, London, United Kingdom; h Department of Medicine, Mayo Clinic, Rochester, Minnesota, USA; i Heart, Vascular, and Thoracic Institute, Cleveland Clinic, Cleveland, Ohio, USA; j Cardiovascular Innovation Research Center, Cleveland, Ohio, USA; k National Institutes of Health, National Heart, Lung, and Blood Institute, Bethesda, Maryland, USA

**Keywords:** dilated cardiomyopathy, cardiovascular magnetic resonance, midwall septal fibrosis, arrhythmia, diffusion tensor imaging

## Abstract

**Background:**

The presence of midwall septal fibrosis (MSF) in dilated cardiomyopathy (DCM) has been shown to be associated with adverse clinical outcomes, but the underlying pathophysiological mechanisms are incompletely understood. We investigated whether MSF associates with a distinct pattern of myocardial microstructural and microvascular abnormalities using advanced cardiovascular magnetic resonance (CMR).

**Methods:**

This was a prospective, multi-referral, single-center study comparing the hearts of patients with a current or prior diagnosis of DCM with and without MSF (“MSF+”/“MSF–”), to a control cohort of a similar age and sex distribution. All underwent single-magnet 3 Tesla CMR, including cardiac diffusion tensor imaging (cDTI), quantitative rest perfusion, and multiparametric tissue characterization. Prespecified analyses compared DCM with controls, and MSF+ with MSF–; secondary analyses included regional septal and within-subject segmental comparisons.

**Results:**

About 121 participants were studied: 34 MSF+ (51±14years; 74% male), 27 MSF– (48±15 years; 63% male), and 60 controls (45±13 years; 58% male). Compared with controls, the DCM cohort demonstrated increased mean diffusivity (MD) (1.49 v 1.43 ×10^−3^mm^2^/s, *p*<0.001) and reduced second eigenvector angle (E2A) (34.7 vs. 40.2°, *p*=0.001), consistent with microstructural abnormality, along with reduced resting myocardial blood flow (rMBF) (0.66 vs. 0.70 mL/g/min, *p*=0.045). Within the DCM cohort, MSF+ patients exhibited increased MD (1.51 vs. 1.46 ×10^−3^mm^2^/s, *p*=0.006) and decreased rMBF (0.64 vs. 0.71 mL/g/min, *p*=0.013) compared to MSF–. Septal analyses demonstrated increased MD, decreased E2A, decreased FA, and reduced rMBF in DCM. Within-patient comparisons showed decreased perfusion in fibrotic segments compared with non-fibrotic myocardium. In exploratory analyses, decreased rMBF was associated with greater ventricular ectopic burden.

**Conclusion:**

Midwall septal fibrosis in DCM identifies a distinct myocardial phenotype characterized by microstructural remodeling and impaired myocardial perfusion, with regional and segmental specificity. These findings provide mechanistic insight into the adverse prognostic associations of MSF and highlight a potential imaging-guided pathway for risk stratification and therapies.

## Graphical abstract

**Figure:**
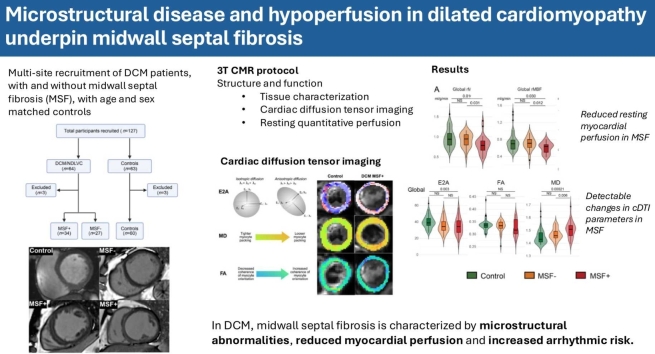

## 1 Introduction

Dilated cardiomyopathy (DCM) is a heterogenous heart muscle disease characterized by left ventricular (LV) systolic dysfunction, with or without LV or biventricular dilatation, occurring in the absence of abnormal loading conditions or significant coronary artery disease [1]. It affects ∼1 in 250 adults [2], and its clinical course is highly variable, ranging from stable ventricular function to progressive heart failure, malignant ventricular arrhythmia and sudden cardiac death (SCD).

An imaging marker that has gained increasing attention is midwall septal fibrosis (MSF), identified on cardiovascular magnetic resonance (CMR) as interventricular septal late gadolinium enhancement (LGE). MSF consists of replacement myocardial fibrosis [3], [4] and is considered to be an irreversible phenomenon [5], [6]. MSF is a common CMR finding in DCM, observed in one-third of cases. Importantly, MSF has been shown to have independent and incremental prognostic value beyond LV ejection fraction (LVEF) for predicting all-cause and cardiovascular mortality, including SCD [7], [8], [9]. As a result, clinicians are increasingly using the presence or absence of MSF to stratify participants with DCM for medical and device therapies, but this has not yet been implemented in international guidelines [10]. Nevertheless, the pathophysiological mechanisms underpinning the pro-arrhythmic potential of MSF in DCM, remain incompletely understood [11].

**Fig. 1:**
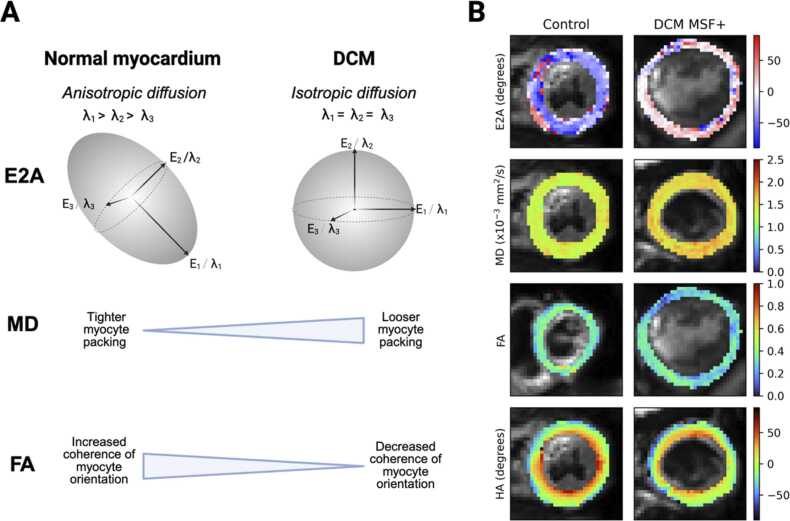
The microstructural abnormalities in DCM with MSF can be detected using cardiac DTI. Panel A: Expected cardiac DTI metrics in normal myocardium and DCM. A diffusion imaging tensor is represented pictorially in the top panel: isotropic diffusion is depicted as a sphere, and anisotropic diffusion is depicted as an ellipsoid. λ denotes the eigenvalue (the magnitude of diffusion), and E denotes the eigenvector (the direction of diffusion). In normal myocardium, water diffusion exhibits an anisotropic pattern, diffusing preferentially along the long axis of densely organized cardiomyocytes. In contrast, DCM is associated with an isotropic diffusion pattern in areas of fibrosis or myocardial disarray. MD is the mean of λ_1,_ λ_2_, and λ_3_ and measured in units of square millimeters per second. MD measures the overall magnitude of diffusion and represents myocyte packing, with increased values reflecting looser cell packing, e.g. interstitial fibrosis, as water has more freedom to diffuse. FA is a scalar value which measures restrictiveness to water diffusion. In normal tissue, cell membranes act as barriers and restrict water diffusion. Therefore, FA is expected to be high in voxels with coherently aligned myocytes with a consistent orientation such as normal tissue. Conversely, FA is expected to be low in voxels with differing myocyte orientations, e.g. in conditions with disorganized cell orientations and increased ECV. The microstructure of the myocardium consists of a branching syncytium of cardiomyocytes embedded in a collagen matrix. These cardiomyocytes aggregate to form laminar secondary structures called ‘sheetlets’. Sheetlet orientation and re-orientation is a key facilitator of LV wall thickening, and the sheetlet orientation can be measured with cDTI as the E2A. Panel B: In this study, DCM patients with midwall septal fibrosis demonstrated increased MD, lower E2A, no difference in FA. HA maps for control and MSF+ subjects are also shown. *DCM* dilated cardiomyopathy, *MSF* midwall septal fibrosis, *MD* mean diffusivity, *FA* fractional anisotropy, *LV* left ventricular, *cDTI* cardiac diffusion tensor imaging, *E2A* secondary eigenvector angle, *HA* helix angle

Cardiac diffusion tensor imaging (cDTI) is a CMR imaging technique that measures the direction and magnitude of restriction to water molecule diffusion within an imaging voxel. In the normal myocardium, such natural restrictions arise from intact cell walls and collagen sheetlets [12]. As water diffuses preferentially along the long axis of cardiomyocytes, cDTI can provide information on the principal orientations of cardiomyocytes and intramyocardial sheetlets [13], and on the myocardium’s microstructural environment at the level of cardiomyocyte organization [14]. By extension, cDTI and its derived biomarkers, the secondary eigenvector angle (E2A), mean diffusivity (MD), and fractional anisotropy (FA) (Fig. 1), can provide novel insights into diseased myocardial states and the pathophysiology underlying cardiomyopathic processes. Models to predict mechanics and arrhythmogenesis in the LV are increasingly incorporating microstructural information, such as cardiomyocyte orientation, as a variable [15], [16], [17].

In this study, we sought to determine whether MSF in DCM marks a distinct myocardial phenotype characterized by microstructural abnormalities and impaired myocardial perfusion.

## 2 Materials and methods

### 2.1 Ethics

This study was approved by the North West Greater Manchester West Research Ethics Committee, the Health Research Authority and Health and Care Research Wales Authority, under reference [21]/NW/0333, approval date 24 November 2021 (protocol number 143656 and IRAS ID: 290788), and is registered under ClinicalTrials.gov ID: NCT05026112. The study conformed to the principles of the Helsinki Declaration, and all participants gave written informed consent. Participants were not compensated for participation.

### 2.2 Recruitment

Between October 2021 and April 2024, this prospective multi-referral, single-center study recruited participants with current or previously diagnosed DCM with or without MSF (“MSF+”, “MSF–” respectively), and healthy volunteers of similar age and sex (referred to as ‘controls’). Patients were recruited from the Heart Failure and Inherited Cardiac Conditions Clinics of three sites: Royal Free Hospital, Barnet Hospital and Chase Farm Hospital, and through direct participant self-referral via The Heart Hive (https://thehearthive.org). The DCM cohort included patients with previously diagnosed DCM, including those with reverse remodeling and recovered ventricular function on therapy (see **Supplementary Material, Table S3** for comparison of cohort characteristics**)**.

DCM was defined by the presence of LV dilatation and global systolic dysfunction (LVEF <50%) by echocardiography or CMR [10]. Participants were recruited without any specification on LVEF and for simplicity, DCM/NDLVC patients are hitherto collectively referred to as the ‘DCM cohort’ (see **Supplementary Material, Table S3** for cohort characteristics**)**. MSF was defined as the presence of midwall septal LGE in a linear, streaky or patchy distribution, visible in at least two cross-sectional imaging planes. All participants underwent next generation sequencing [18] with the 48-DCM-gene panel performed at the same laboratory, and the interpretation of the pathogenicity of genetic variants followed the American College of Medical Genetics guidelines [19]. All patients also underwent supine resting 12-lead ECG on the same day as CMR, as well as 24-hour ambulatory ECG monitoring within 6 months of being recruited. Ambulatory ECGs were analyzed to measure the burden of ventricular ectopy (VE), nonsustained ventricular tachycardia (NSVT), other ventricular arrhythmias (e.g. ventricular bigeminy/trigeminy, couplets, etc.), supraventricular ectopy and atrial fibrillation. NSVT was defined as runs of beats arising from the ventricles with duration between 3 beats and 30 seconds and with cycle length of <600 ms (>100 beats per minute) [20]. For the purpose of the exploratory VE/NSVT analysis, VE burden of >0.1% of total beats, or any NSVT within the 24-hour tape was counted as ‘1’, otherwise '0’.

Controls were recruited through advertisement via posters in collaborating hospitals and from staff at University College London. Controls reported no known significant medical history or cardiac disease, no family history of cardiomyopathy or SCD, were not professional or high-endurance athletes, and were not on any cardiac medications apart from lipid-lowering therapy.

Exclusion criteria for all participants included renal insufficiency with estimated glomerular filtration rate of <30 mL/min/1.73 m^2^, claustrophobia, needle-phobia precluding cannulation for the administration of gadolinium-based contrast agent (GBCA) and other general contraindications to CMR (e.g., pregnancy, morbid obesity). Participants with cardiac implantable electronic devices (excluding implantable loop recorders) were also excluded. Participants with a history of significant coronary artery disease (defined as flow-limiting coronary disease on invasive angiography or computerized tomography coronary angiography, or history of myocardial infarction or coronary artery bypass grafting) or infarct-pattern LGE were excluded, but DCM patients and healthy controls with small volume right ventricular insertion point LGE were included. Patients with a history of permanent or paroxysmal atrial fibrillation were not excluded from the study.

### 2.3 CMR imaging protocol

CMR was performed at the University College London Bloomsbury Centre for Clinical Phenotyping using a single 3-Tesla MR system (Siemens Magnetom Prisma, Siemens Healthineers, Erlangen) with an 18-channel phased-array chest coil and spine array (up to 24-elements) equipped with Gadgetron (Linux box, 24 cores). Standard long- and short-axis cine imaging was performed, using breath-held, balanced steady-state free precession pulse sequences as previously described [21], with the following parameters: flip angle 50°; repetition time 29.1; echo time 1.25/2.9 ms; slice-gap 2.0 mm; field of view 380x285 mm; matrix size 256×140; reconstructed voxel size 1.5×1.5×8.0; calculated cardiac phases 30. Basal, mid and apical co-registered pre- and post-GBCA short-axis T1 and T2 maps were generated using free-breathing multiparametric saturation recovery single-shot acquisition (mSASHA) protocol, as previously described [22]. cDTI data were obtained using a free-breathing, second-order motion-compensated single-short spin echo planar imaging sequence with the following acquisition parameters: repetition time/echo time 77 ms/3 R-R intervals, field of view 320 × 121 mm^2^, matrix size 138 × 52, in-plane resolution 2.3 × 2.3 mm^2^, 8 mm slice thickness, 8 mm inter-slice gap, and partial Fourier = 7/8 [23], as previously reported. The trigger delay was adjusted based on the cine data for each subject so that k-zero was acquired at ∼85% of the end-systolic phase. Each cDTI data set comprised b-values of 100 s/mm^2^ (3 diffusion-weighted [DW] directions, 12 repetitions), and 450 s/mm^2^ (30 DW directions, 6 repetitions). The acquisition parameters were consistent with previous recommendations [24].

Quantitative rest perfusion imaging was acquired using a dual sequence approach as previously described [25], that automatically reconstructed in-line rest perfusion maps [26]. Peripheral and central aortic blood pressure using an oscillometric blood pressure machine were recorded at the time of the rest perfusion acquisition to permit rate pressure product (RPP = heart rate x central systolic blood pressure) normalization of resting myocardial blood flow (rMBF_N_). The second bolus of GBCA was injected approximately 5 minutes prior to LGE acquisition. LGE imaging utilized a phase-sensitive inversion recovery technique 10 minutes after the GBCA injection (gadoterate meglumine, 0.1 mmol/kg body weight [Dotarem], Guerbet, Villepinte, France). Post-GBCA T1 maps were acquired 15 min after GBCA injection using the same slice location and field-of-view as the pre-GBCA images, thus generating post-GBCA T1 maps.

### 2.4 Data analysis

#### 2.4.1 Primary analyses and study cohorts

Participants were categorized into three prespecified groups: controls, DCM patients without midwall septal fibrosis (MSF−), and DCM patients with midwall septal fibrosis (MSF+). The primary analyses comprised (1) comparison of the entire DCM cohort with controls to define disease-associated myocardial abnormalities; and (2) comparison of MSF+ and MSF− patients to assess the impact of midwall septal fibrosis as a phenotypic stratifier within DCM.

For the analyses, investigators were blinded to participant groups and clinical data to the greatest extent feasible. While the investigator performing CMR volumetric and LGE analysis could visualize the myocardium during quantification, cDTI post-processing was conducted using fully pseudonymized datasets with no access to patient group, other CMR results, or clinical outcomes.

#### 2.4.2 Prespecified secondary and exploratory analyses

To contextualize the phenotypic effects of MSF, secondary comparisons were performed between MSF+/– subgroups and matched controls, allowing assessment of graded abnormalities across control, MSF−, and MSF+ groups.

Given that midwall septal fibrosis is predominantly found in the basal and mid septum, regional analyses were performed focusing the septal segments (AHA segments 2, 3, 8, and 9) in the DCM cohort.

To isolate local myocardial effects of focal fibrosis independent of inter-individual variability, a secondary mechanistic analysis was undertaken in MSF+ cohort, which we termed “within-patient segmental analysis”. Within each MSF+ participant, myocardial segments with the highest fibrosis burden were classified as fibrotic myocardium (“LGE+”), and compared to segments with the lowest fibrosis burden, classified as non-fibrotic myocardium (“LGE–”).

Finally, exploratory analyses were undertaken. A multiparametric correlation analysis was performed to examine associations between ventricular ectopy burden and myocardial microstructural, perfusion, and tissue characterization parameters. In addition, to contextualize microstructural and microvascular abnormalities according to disease severity, DCM patients were stratified by left ventricular ejection fraction into heart failure with reduced ejection fraction (HFrEF; LVEF ≤40%), mildly reduced ejection fraction (HFmrEF; LVEF >40 to <50%), and preserved ejection fraction (HFpEF; LVEF ≥50%) [27], and global myocardial imaging metrics were compared across LVEF spectra.

#### 2.4.3 Volumetric analysis

All CMR analytics and post-processing were conducted in CVI42 version 5.13.7 (Circle Cardiovascular Imaging Inc, Calgary, Canada) by the same Level II accredited reader (F.C.). Left and right ventricular volumes and ejection fractions and LV mass were calculated from semi-automatically drawn end-diastolic and end-systolic contours on short-axis cines, excluding the papillary muscles and trabeculae using commercially available software cvi42. LV maximal wall thickness was measured as the thickest end-diastolic wall thickness measurement on the LV cine short-axis stack.

#### 2.4.4 Tissue characterization

T1 and T2 maps were analyzed using CVI42 software and manual extracellular fluid volume (ECV) maps were also generated. Manual epicardial and endocardial contours applied to the basal, mid, and apical ventricular short-axis slices were automatically eroded inwards by a 20% offset of the local wall thickness to avoid confounding by blood partial volume effects at the blood myocardial interface. Maps were then segmented according to American Heart Association (AHA) segment model [28] excluding segment 17. For blood T1 analysis, a region of interest (ROI) was drawn in the central LV blood pool in the three short-axis slices, avoiding papillary muscles or trabeculae. The contours and blood pool ROIs were copied from the native T1 short-axis slices onto the corresponding post-GBCA T1 images. Manual ECV mapping measurements were then calculated according to the formula ECV= [Δ(1/T1myo)/ Δ(1/T1blood)*((1-Hct)] [29].

#### 2.4.5 Quantification of late gadolinium enhancement

Two experienced readers (CMR Level III, G.C. & CMR Level II, F.C.) independently analyzed the CMR LGE data. The presence and pattern of LGE were adjudicated by consensus. For discordant rulings, our protocol stipulated that LGE data would be independently reviewed by a third reader (CMR Level III, JCM) for a consensus ruling. LGE quantification was carried out using the tissue signal intensity module in CVI42. Short-axis LGE images were manually segmented for epicardial and endocardial borders. The LGE extent was then measured semi-automatically by signal thresholding using the 5-standard deviation (SD) method [30]. Absolute LGE mass (expressed in grams) and the relative enhanced LGE area per segment (expressed as %) were calculated per AHA segment.

#### 2.4.6 First-pass resting myocardial perfusion

Inline automatic reconstruction and post-processing of resting first pass perfusion data were implemented with the Gadgetron software framework, as previous described [31]. Automatic segmentation of the LV cavity and myocardium was performed by an artificial intelligence tool [26], excluding myocardial fat and papillary muscles. RPP-normalized global and segmental mean MBF were calculated inline from the perfusion maps, where each pixel encoded MBF in mL/g/min. Each LV segment was further subdivided into subendocardial (inner 50%) and subepicardial (outer 50%) regions. Basal segments that included LV outflow tract or other significant artifact were excluded from subsequent analysis. Resting MBF (rMBF) was calculated per segment and averaged across all 16-segments to represent mean global values.

#### 2.4.7 Cardiac DTI

cDTI images were processed as described previously [23] using an in-house developed toolbox. Registration was performed for each slice individually using SimpleITK with Mutual Information as a metric, calculated within a rectangular mask. An Outlier Rejection technique was used to semi-automatically remove misregistered or severely motion-corrupted images [32]. The diffusion tensor was fitted using weighted linear least squares regression to the data, and diffusion metrics such as FA, MD, and E2A were calculated for each voxel. The LV in each slice was segmented manually using the same toolbox. cDTI data were generated for each myocardial segment as well as a global value. Regions of the LV corrupted by susceptibility-related distortion or local residual artifacts were excluded from subsequent calculations of the global/segmental metrics.

To ensure there was no difference in the signal-to-noise ratio (SNR) between control and DCM groups that could impact the cDTI results, 10 participants per group were selected at random. SNR was derived from the measurement of the mean signal intensity of a manually placed ROI in the mid-myocardial septum, and from the measurement of the standard deviation of pixel intensity from air (in the lung field) in the same image.

### 2.5 Statistical analysis

Statistical analysis was performed using RStudio (version 2023.12.1). Distribution of data was assessed through visual inspection of histograms and QQ plots and formally tested using the Shapiro-Wilk test. Categorical variables, reported as counts (%), were compared using Chi-squared or Fisher’s exact test as appropriate. Continuous variables are reported as mean±SD if normally distributed, or median (interquartile range) if non-normally distributed. Normally distributed continuous variables were compared using the unpaired Student *t*-test or analysis of variance (ANOVA), while non-normally distributed continuous variables were compared using the Wilcoxon rank-sum test. Continuous variables were analyzed using linear regression models with group as a three-level categorical factor (controls, MSF– and MSF+). For the secondary, within-subject segmental analysis, for each MSF+ patient, myocardial segments with the highest fibrosis burden were classified as fibrotic myocardium (“LGE+”), and segments with the lowest fibrosis burden were classified as remote non-fibrotic myocardium (“LGE–”). To compare clinical CMR and cDTI parameters between LGE+ and LGE– segments within subjects, linear mixed effect models (LMM) were fitted, with fibrosis status (LGE presence) as the fixed effect and Patient ID as a random effect to account for intra-patient clustering. Model residuals were examined for normality to validate model assumptions. Due to the skewed distribution of CMR parameters, generalized linear models (GLMs) with a gamma distribution and log link were used for univariate analyses with continuous outcome variables (e.g., cDTI, rMBF). For univariate analyses with a binary outcome (presence of ventricular arrhythmia), GLMs with binomial distribution and logit link (i.e., logistic regression) were applied. Variables significantly associated with VE/NSVT presence in univariate GLMs were considered for inclusion in multivariable logistic regression models. Predictor variables were exponentiated using the “parameters” package in R to obtain exponentiated *β* coefficients. Correlations between continuous variables were assessed using Pearson’s or Spearman’s correlation coefficients, as appropriate, and visualized using heatmaps.

All statistical tests were two-tailed, and *p* < 0.05 was considered statistically significant.

## 3 Results

**Fig. 2:**
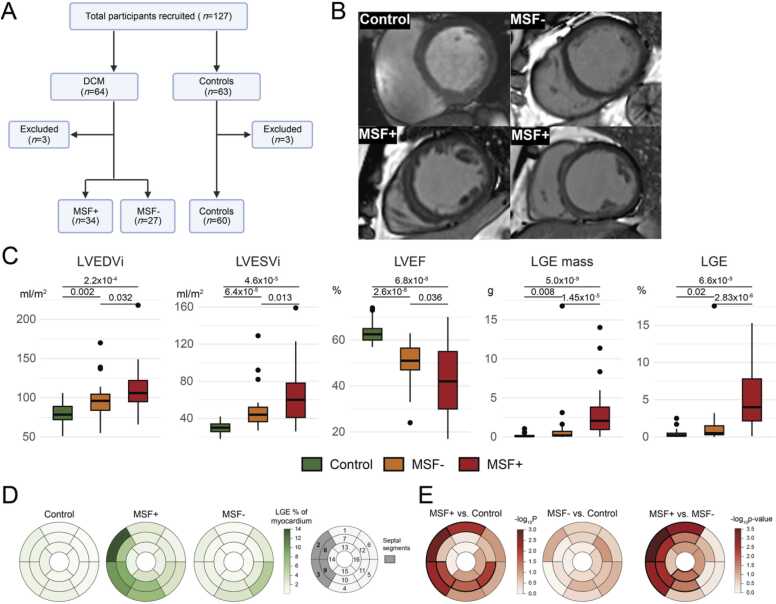
Study overview and clinical characterization of DCM cohort and controls. **A**) CONSORT flow diagram of study design. **B**) Representative mid-ventricular short-axis CMR images of control, DCM without mid-wall septal fibrosis (MSF–) and DCM with mid-wall septal fibrosis (MSF+) from the study cohort. **C**) Box and whisker plots for global LV features in DCM cohort, stratified by MSF status (MSF+ red; MSF– orange) and controls (green). **D**) Bullseye plot of percentage of LGE within each myocardial segment in MSF+, MSF– and controls. Darker colors indicate larger values. **E**) Bullseye plot of -log_10_*p*-values for between group comparisons of LGE percentage in myocardial segments, with darker colors indicating smaller *p*-values. Statistically significant segments (*p*-value<0.05) are bordered in a thick edge. *DCM* dilated cardiomyopathy, *CMR* cardiovascular magnetic resonances, *MSF* midwall septal fibrosis, *LV* left ventricular, *LGE* late gadolinium enhancement

A total of 127 participants were recruited (Fig. 2**A**), consisting of 64 patients in the DCM cohort and 63 controls. Three patients and two controls were unable to complete CMR, and one control was found to have newly diagnosed atrial fibrillation, leaving a final set of 61 patients and 60 controls for analysis. Of the 61 DCM patients, 34 were MSF+ and 27 MSF–. There were no discordant MSF rulings between the first two readers, meaning that consensus review by the third reader was not required (representative short-axis images in Fig. 2**B**). Regarding data completeness and quality: for the cDTI analysis, 16 participants (9 control and 7 DCM patients) did not have cDTI data available due to artifact. Out of 1680 myocardial segments available for cDTI analysis, 133 segments (7.9%) contained artifact so were excluded. For the perfusion data, three DCM patients did not have perfusion data available due to artifact and incorrect slice positioning. Out of 1888 myocardial segments available for perfusion analysis, 72 segments were excluded (3.8%). There was no difference in the SNR between the control and DCM groups.

Clinical and demographic characteristics of study participants are summarized in Table 1 and CMR parameters in Table 2. MSF+ and MSF– participant groups were similar on the basis of age, sex and BSA (all *p* > 0.05) but MSF+ patients had reduced LVEF (*p*=0.037), LV dilation (*p*<0.001), indexed mass (*p*=0.030), LGE mass (*p*<0.001), and percentage LGE myocardial burden (*p*<0.001, Fig. 2**C**). There were no significant differences in right ventricular ejection fraction, right ventricular volumes and left atrial volumes observed between MSF+ and MSF–. As expected, MSF+ had significantly more LGE than both MSF– (*p*<0.001) and controls (*p*<0.001, Figs. 2**D** and 2**E**). As expected for a 3-Tesla CMR study, a variable extent of superior and inferior RV insertion point LGE was measurable in most study participants, but these LGE volumes were trivial in controls (median LGE 0.10 g) and only marginally higher in MSF– (median 0.20 g). MSF+ had greater VE burden on ambulatory 24-hour ECG monitoring when compared to MSF**–** participants. 58% of VEs detected in MSF+ were polymorphic when compared to 50% in MSF– patients. NSVTs were rare and only detected in 4 and 3 MSF+/– patients, respectively (*p*=NS). Atrial fibrillation was uncommon and was detected in 3 MSF+ and 2 MSF– patients.

**Table 1:** Baseline clinical and demographic characteristics of study cohorts

**Characteristic**	**DCM cohort** ***n*****=61**	**MSF+** ***n*****=34**	**MSF–** ***n*****=27**	**Controls** ***n*****=60**	***p*****-value** **DCM cohort vs.** **controls**	***p*****-value** **MSF+ vs. MSF–**
Age, years	49.8±14.3	51.3±13.8	47.9±15.1	44.8±12.7	**0.048**	0.366
Male sex, counts (%)	42 (69%)	25 (74%)	17 (63%)	35 (58%)	0.260	0.415
BSA, m^2^	1.93±0.24	1.92±0.25	1.94±0.20	1.83±0.20	**0.026**	0.780
*NYHA functional class*						
NYHA I	27 (44%)	18 (53%)	19 (70%)			0.387
NYHA II	20 (33%)	12 (35%)	8 (30%)			1.000
NYHA III	1 (2%)	1 (3%)	0			1.000
NYHA IV	3 (5%)	3 (9%)	0			0.248
*Genetic variants*						
*MYH7* (P/LP)	1 (1.6%)	0	1 (3.7%)			0.443
*TTN* (P/LP)	8 (13%)	7 (21%)	1 (3.7%)			0.059
*BAG3* (P/LP)	1 (1.6%)	1 (3%)	0			1.000
*FLNC* (P/LP)	1 (1.6%)	1 (3%)	0			1.000
*DSP* (P/LP)	1 (1.6%)	1 (3%)	0			1.000
*VUS*	7 (11%)	4 (12%)	3 (11%)			1.000
Gene elusive	42 (69%)	20 (59%)	22 (82%)			**0.036**
*Clinical parameters*						
Peripheral SBP, mmHg	124±25	128±8	125±15	124±16	0.756	0.554
Peripheral DBP, mmHg	78±13	79±13	79±11	80±12	0.236	0.972
Central SBP, mmHg	120±19	122±8.1	121±10	118±7	0.737	0.623
Central DBP, mmHg	75±12	76±10	76±9	76±9	0.488	0.319
Smoking history, counts (%)	24 (39%)	15 (44%)	9 (33%)	2 (3%)	**<0.001**	0.596
Hypertension, counts (%)	16 (26%)	10 (29%)	6 (22%)			0.572
Dyslipidemia, counts (%)	15 (25%)	7 (21%)	8 (30%)	6 (10%)	0.154	0.551
Atrial fibrillation, counts (%)	8 (13%)	4 (12%)	4 (15%)			0.722
T2DM, counts (%)	9 (15%)	8 (24%)	1 (4%)			**0.033**
*Medications*						
ACEi/ARB	23 (38%)	11 (32%)	12 (44%)			0.427
Beta blocker	50 (82%)	30 (88%)	20 (74%)			0.190
ARNI	28 (46%)	18 (53%)	10 (37%)			0.302
Diuretics	14 (23%)	12 (35%)	2 (7%)			**0.014**
SGLT2i	28 (46%)	19 (56%)	9 (33%)			0.121
MRA	28 (46%)	19 (56%)	9 (33%)			0.121
Lipid lowering agents	17 (28%)	12 (35%)	5 (19%)	6 (10%)	**0.035**	0.165
Antiarrhythmics	3 (5%)	0	3 (11%)			0.080
Anticoagulants	14 (23%)	9 (26%)	5 (19%)			0.549
*12-Lead ECG*						
HR, bpm	65 (58–72)	65 (59–72)	65 (57–70)	63 (56–70)	0.645	0.467
LBBB, counts (%)	9 (15%)	4 (12%)	5 (19%)	0		0.722
RBBB, counts (%)	0	0	0	0		
QRS duration, ms	93±14	90±12	98±14	87±11	**0.007**	0.058
PR interval, ms	118±16	116±16	121±15	108±20	**0.003**	0.309
QTc, ms	425±30	430±30	418±29	404±23	<0.001	0.154
*Ambulatory ECG monitoring*						
VE burden, % of total beats	1.7±3.4	2.0±3.5	1.1±3.2			**0.017**
SVE burden, beats/24 hours	3 (0–20)	4 (1–20)	2 (0–16)			0.549

Results are reported as mean±SD, median (quartile 1-quartile 3) or n (%). Difference test *p*-values are obtained using student *t*-test or Wilcoxon rank-sum test for continuous variables and Chi Square or Fisher’s exact test for categorical variables as appropriate

*ACEi* angiotensin converting enzyme inhibitor, *ARB* angiotensin II receptor blocker, *ARNI* angiotensin receptor/nephrolysin inhibitor, *BAG3* Bcl2-associated athanogene 3, *BSA* body surface area, *DBP* diastolic blood pressure, *DCM* dilated cardiomyopathy, *DSP* desmoplakin, *FLNC*, filamin C, *MRA* mineralocorticoid receptor antagonist, *MSF* midwall septal fibrosis, *MYH7* β-myosin heavy chain 7, *NSVT* nonsustained ventricular tachycardia, *NYHA* New York Heart Association, *P/LP* pathogenic/likely pathogenic, *SBP* systolic blood pressure, *SGLT2* sodium/glucose co-transporter II inhibitor, *SVE* supraventricular ectopy, *T2DM* type 2 diabetes mellitus, *TTN* titin, *VE* ventricular ectopy, *VUS* variant of uncertain significance

**Table 2:** CMR characteristics in the cohorts

**Characteristic**	**DCM cohort** **(*****n*****=61)**	**MSF +** **(*****n*****=34)**	**MSF–** **(*****n*****=27)**	**Controls** **(*****n*****=60)**	***p*****-value** **DCM** ***vs.*****controls**	***p*****-value** **MSF+*****vs.*****MSF–**
*Structure and function*						
LVMassi (g/m^2^)	67±17	71±51	62±13	51±9	**<0.001**	**0.030**
LVEDVi (mL/m^2^)	107±39	107 (96–123)	96 (84–105)	80±12	**<0.001**	**0.033**
LVESVi (mL/m^2^)	62±42	62 (41–83)	44 (37–52)	30±6	**<0.001**	**0.014**
LVEF, %	49 (35–58)	42 (30–55)	51 (47–57)	63 (60–65)	**<0.001**	**0.037**
RVEDVi (mL/m^2^)	96±25	98±29	93±20	89±14	**<0.001**	0.679
RVESVi (mL/m^2^)	51±24	55±30	46±14	38±7	**<0.001**	0.196
RVEF (%)	47±12	45±15	51±7	58±4	**<0.001**	0.092
LAVi (mL/m^2^)	43 (30–52)	42 (29–53)	44 (31–50)	37 (31–44)	**0.011**	0.988
*Tissue characterization*						
Native global T1^§^, ms	1549±53	1550±59	1549±46	1520±37	**0.001**	0.929
Global T2^§^, ms	38.6 (37.5–40.1)	39.3 (37.7–41.7)	38.2 (37.2–39.2)	37.2 (36.6–39.1)	**0.001**	0.035
LGE mass, g	0.9 (0.2–2.9)	2.2 (1.0–4.4)	0.2 (0.1–0.7)	0.1 (0.0–0.2)	**<0.001**	**<0.001**
LGE volume, %	1.9 (0.5–4.9)	4.1 (2.2–7.8)	0.5 (0.3–1.5)	0.2 (0.1–0.5)	**<0.001**	**<0.001**
ECV, %	26.9±3.8	27.7±4.2	25.8±2.9	25.2±3.3	**0.017**	0.059
*Resting myocardial perfusion*						
	**DCM cohort** **(*****n***=58)*	**MSF+** **(*****n***=32)*	**MSF–** **(*****n***=26)*	**Controls** ***(n*****=60)**	***p*****-value** **DCM** ***vs.*****controls**	***p*****-value** **MSF+*****vs.*****MSF–**
Global mean rMBF, mL/g/min	0.66 (0.56–0.73)	0.64 (0.50–0.68)	0.71 (0.61–0.80)	0.70 (0.57–0.78)	**0.045**	**0.013**
Global mean rMBF_N_, mL/g/min	0.85 (0.74–0.99)	0.79 (0.67–0.90)	0.95 (0.80–1.06)	0.94 (0.80–1.09)	**0.014**	**0.032**
Mean endocardial rMBF, mL/g/min	0.71 (0.60–0.78)	0.69 (0.54–0.74)	0.76 (0.65–0.84)	0.75 (0.63–0.86)	0.064	**0.017**
Mean endocardial rMBF_N_, mL/g/min	0.92 (0.79–1.08)	0.89 (0.72–0.98)	1.01 (0.85–1.17)	1.02 (0.84–1.22)	**0.034**	0.053
Mean epicardial rMBF, mL/g/min	0.61 (0.50–0.700	0.60 (0.46–0.64)	0.67 (0.58–0.72)	0.66 (0.54–0.75)	**0.029**	**0.013**
Mean epicardial rMBF_N_, mL/g/min	0.79 (0.67–0.92)	0.75 (0.61–0.83)	0.88 (0.74–0.96)	0.88 (0.75–1.02)	**0.005**	**0.024**
*Cardiac DTI*						
	**DCM cohort** **(*****n*****=54)**^**†**^	**MSF+** **(*****n*****=30)**^**†**^	**MSF–** **(*****n*****=24)**^**†**^	**Controls** ***(n*****=51)**^**†**^	***p*****-value** **DCM** ***vs.*****controls**	***p*****-value** **MSF+*****vs.*****MSF–**
Global MD, x10^−3^mm^2^/s	1.49 (1.45–1.53)	1.51 (1.47–1.54)	1.46 (1.44–1.49)	1.43 (1.41–1.48)	**<0.001**	**0.006**
Global FA	0.33 (0.30–0.35)	0.32 (0.30–0.36)	0.33 (0.32–0.35)	0.34 (0.33–0.34)	0.150	0.398
Global E2A, degrees	34.7±9.5	34.0±10.3	35.5±8.5	40.2±7.6	**0.001**	0.573
*Septal analysis – AHA segments 2, 3, 8, 9*						
Septal MD, 10^−3^ mm^2^/s	1.49 (1.44–1.52)	1.51 (1.48–1.55)	1.44 (1.42–1.49)	1.44 (1.41–1.48)	**0.003**	**0.003**
Septal FA	0.31 (0.27–0.34)	0.30 (0.27–0.34)	0.32 (0.28–0.34)	0.35 (0.32–0.38)	**0.002**	0.566
Septal E2A, degrees	34.5±11.7	32.6±12.2	36.7±7.7	40.1±8.8	**0.007**	0.695
Septal mean rMBF, mL/g/min	0.61 (0.52–0.69)	0.60 (0.49–0.66)	0.64 (0.56–0.70)	0.67 (0.55–0.75)	**0.032**	0.517
Septal mean rMBF_N_, mL/g/min	0.82±0.26	0.77 (0.58–0.89)	0.88 (0.72–1.00)	0.92±0.22	**0.021**	0.332
Septal native T1, ms	1576±51	1578±54	1574±49	1543±43	**<0.001**	0.751
Septal T2, ms	39.0 (37.7–40.9)	40.2 (38.2–43.1)	38.5 (37.6–39.4)	37.5 (37.0–39.6)	**0.003**	**0.021**
Septal ECV, %	27.3±4.4	28.6±4.8	25.7±3.1	25.3±3.6	**0.011**	**0.012**

Values are reported as mean±SD or median (quartile 1-quartile 3). Unpaired Student's t-test was used for normally distributed values and Wilcoxon rank-sum test for non-normally distributed variables

*E2A* second eigenvector angle, *ECV* extracellular volume, *FA* fractional anisotropy, *LAVi* indexed left atrial volume, *LGE* late gadolinium enhancement, *LV* left ventricular, *LVEF* left ventricular ejection fraction, *LVEDVi* left ventricular end-diastolic volume, indexed, *LVESVi* indexed left ventricular end systolic volume, *LVMassi* left ventricular mass indexed, *rMBFN* resting myocardial blood flow normalized to rate pressure product, *MD* mean diffusivity, *MWT* maximum wall thickness, *RPP* rate pressure product, *RVEF* right ventricular ejection fraction, *RVEDVi* indexed right ventricular end diastolic volume, *RVESVi* indexed right ventricular end systolic volume, *ACEi* angiotensin converting enzyme inhibitor, *ARB* angiotensin II receptor blocker, *ARNI* angiotensin receptor/nephrolysin inhibitor, *BAG3* Bcl2-associated athanogene 3, *BSA* body surface area, *DBP* diastolic blood pressure, *DCM* dilated cardiomyopathy, *DSP* desmoplakin, *MRA* mineralocorticoid receptor antagonist, *MSF* midwall septal fibrosis, *MYH7* β-myosin heavy chain 7, *NSVT* nonsustained ventricular tachycardia, *NYHA* New York Heart Association, *P/LP* pathogenic/likely pathogenic, *SBP* systolic blood pressure, *SGLT2* sodium/glucose co-transporter II inhibitor, *SVE* supraventricular ectopy, *T2DM* type 2 diabetes mellitus, *TTN* titin, *VE* ventricular ectopy, *VUS* variant of uncertain significance

^§^Normal range for T1 mSASHA at our institution is 1520 ±37 ms; T2 mSASHA=37.2ms (36.6–39.1) *3 DCM patients did not have myocardial perfusion data (2 MSF+,1 MSF**–)**

†16 participants did not have cDTI data (9 control and 7 DCM, comprising of 4 MSF+ and 3 MSF**–**)

Pathogenic/likely pathogenic (P/LP) variants and variants of unknown significance were identified in 31% (19/61) in the DCM cohort. Titin (*TTN*) variants were the most common overall (13% [8/61] of all DCM patients and were more frequently observed in the MSF+ group (21% [7/34] vs. 3.7% [1/27] in MSF–, *p*=0.059). Other P/LP variants were rare (Table 1, and **Supplementary Materials, Table S1**). The MSF– group had a higher proportion of gene-elusive cases (82% [22/27] vs. 59% [20/34] in MSF+, *p*=0.036).

### 3.1 Global myocardial abnormalities in DCM

To define disease-associated myocardial abnormalities, comparison of the DCM cohort (n=61) vs. control (n=60) was undertaken. For the cDTI analysis, the DCM cohort (n=61) demonstrated global microstructural abnormalities: E2A was decreased in the DCM cohort (*p*=0.001), while MD was increased (*p*<0.001). FA did not differ significantly between groups (Table 2). For the perfusion analysis, global rMBF was reduced in DCM patients compared with controls (*p=*0.045), with a more pronounced difference following RPP normalization (*p*=0.014) (Table 2). Lastly, in terms of tissue characterization, DCM patients demonstrated increased global native T1, T2, and ECV compared with controls (*p*=0.001, *p*=0.001, and *p*=0.017 respectively), consistent with diffuse myocardial remodeling (Table 2).

MSF+ vs. MSF–: the impact of midwall septal fibrosis

In order to assess the impact of MSF as a phenotypic stratifier within the DCM cohort, comparison of MSF+ (n=34) and MSF− (n=27) patients was undertaken. In the cDTI analysis, MSF+ patients demonstrated significantly increased global MD compared with MSF− patients (*p*=0.006). Global E2A and FA did not differ significantly between MSF + and −. In terms of myocardial perfusion, global rMBF was significantly reduced in MSF+ compared with MSF− patients (*p*=0.013), with differences persisting after RPP normalization (*p*=0.032). In terms of tissue characterization, only global T2 time were significantly increased in MSF+ compared with MSF− patients (*p*=0.035), while global ECV and T1 did not differ.

### 3.2 Comparison of MSF subgroups with controls

**Fig. 3:**
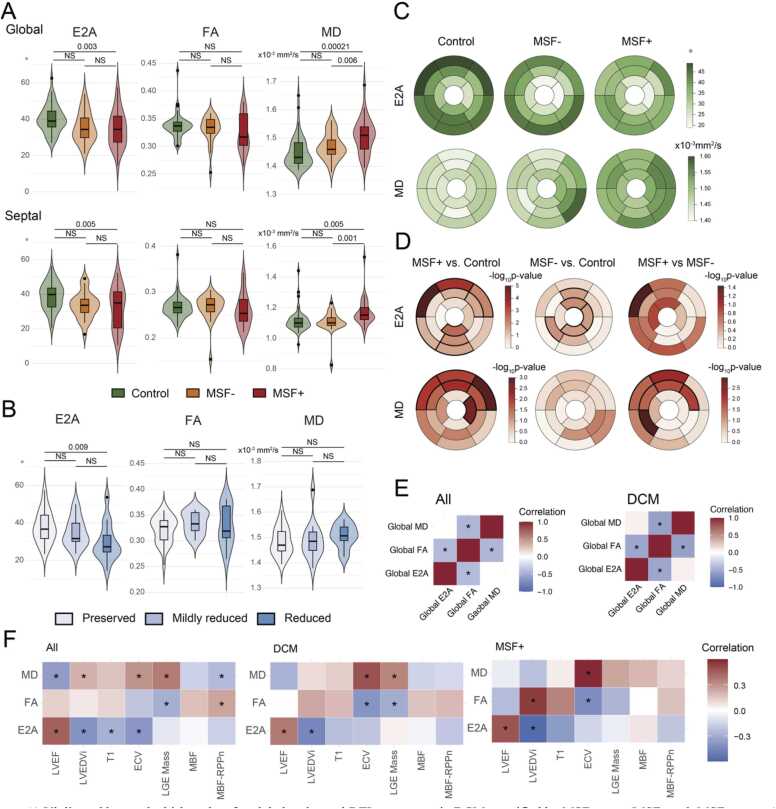
Microstructural characterization of the DCM cohort by cDTI. **A**) Violin and box and whisker plots for global and septal DTI parameters in DCM, stratified by MSF status (MSF+ red; MSF– orange) and all controls (green). The *p*-value represents the comparison between the MSF+/- group and their control group. **B**) Violin and box and whisker plots for global and septal DTI parameters in DCM, stratified by LV systolic function (severely reduced, LV ejection fraction [LVEF] <40% [darkest blue]; mildly reduced, LVEF 41%–49%; and preserved, LVEF >50% [lightest blue]). **C**) Bullseye plot of mean E2A and MD values in myocardial segments in MSF+, MSF–and controls, with darker colors indicating larger values. **D**) Bullseye plot of -log_10_*p*-values for between group comparisons of E2A and MD values in myocardial segments, with darker colors indicating smaller *p*-values. Statistically significant segments (*p*-value<0.05) are bordered in a thick edge. **E**) Correlation heat map between mean global DTI parameters in all participants (left) and those with DCM (right). Asterix indicates significant correlations (*p*-value<0.05). **F**) Correlation heat maps of global LV features with mean global DTI parameters in all participants (left), those with DCM (center), and MSF+ alone (right). Asterix indicates significant correlations (*p*-value<0.05). *DCM* dilated cardiomyopathy, *MSF* midwall septal fibrosis, *MD* mean diffusivity, *LV* left ventricular, *cDTI* cardiac diffusion tensor imaging, *E2A* secondary eigenvector angle

MSF subgroups were compared to controls to explore the grading of abnormalities across control, MSF−, and MSF+ groups (Figs. 3**A and** 4**A**).

**Fig. 4:**
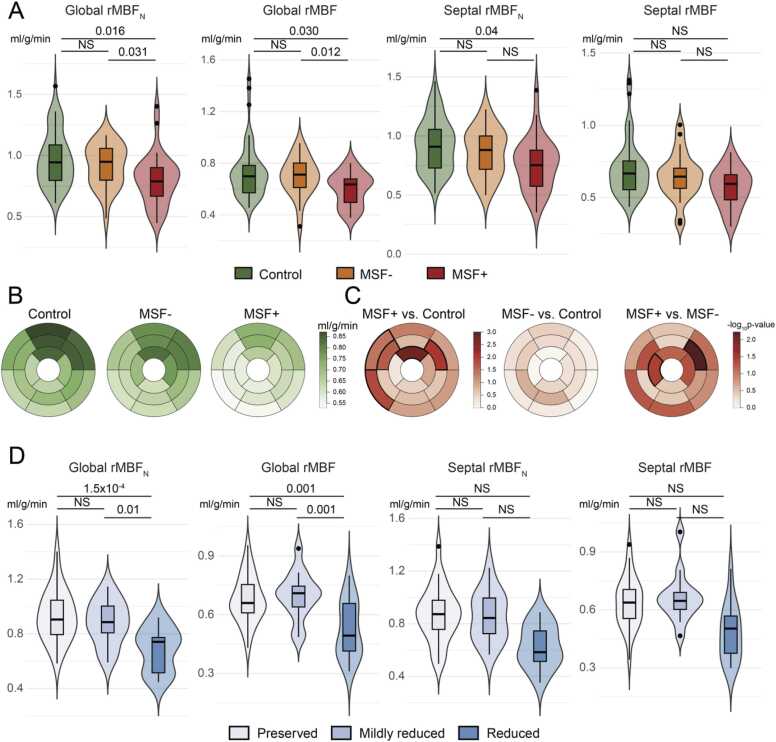
Microvascular characterization of DCM cohort by resting myocardial blood flow. **A**) Violin and box and whisker plots for global and septal resting myocardial blood flow in MSF+ (red), MSF– (orange) and all controls (green). The *p*-value represents the comparison between the MSF+/- group and their controls. **B**) Bullseye plot of absolute resting myocardial blood flow in myocardial segments in MSF+, MSF–and controls, with darker colors indicating larger values. **C**) Bullseye plot of -log_10_*p*-values for between group comparisons of myocardial blood flow in myocardial segments, with darker colors indicating smaller *p*-values. Statistically significant segments (*p*-value<0.05) are bordered in a thick edge. **D**) Violin and box and whisker plots for global and septal myocardial blood flow in DCM, stratified by LV systolic function (severely reduced, LVEF <40% [darkest blue]; mildly reduced, LVEF 41–49%; and preserved, LVEF >50% [lightest blue]). *DCM* dilated cardiomyopathy, *MSF* midwall septal fibrosis, *LVEF* left ventricular ejection fraction

MSF+ patients exhibited widespread differences across microstructural, perfusion, and tissue characterization metrics compared to control (Table 3). Specifically, MSF+ had increased global MD (*p*=0.001), decreased E2A (*p*=0.003), decreased global myocardial perfusion (*p*=0.018), and increased global and septal T1, T2, and ECV (all *p*<0.05). In contrast, MSF− patients demonstrated only isolated increase in native T1 (*p*=0.04) when compared to controls, with no significant differences in cDTI metrics or perfusion metrics (Table 3), suggesting that the global myocardial abnormalities observed in the DCM cohort are driven, at least in part, by the presence of midwall septal fibrosis.

**Table 3:** CMR metrics in the MSF+ and MSF– groups vs. their controls

**CMR metric**	**MSF+** **(n=34)**	**Controls to MSF+** **(n=28)**	***p*****-value** **MSF+*****vs.*****controls**	**MSF–** **(n=27)**	**Controls to MSF–** **(n=27)**	***p-*****value** **MSF–*****vs.*****controls**	***p*****-value MSF +*****vs.*****MSF –**
*Tissue characterization*							
Global native T1, ms	1550±59	1506±34	**<0.001**	1549±46	1525±36	**0.040**	0.929
Global T2, ms	39.3 (37.7–41.7)	36.9 (36.6–38.8)	**<0.001**	38.2 (37.2–39.2)	37.0 (36.3–38.7)	0.080	0.035
Absolute LGE mass, g	2.2 (1.0–4.4)	0.1 (0.1–0.2)	**<0.001**	0.2 (0.1–0.7)	0.1 (0.1–0.2)	**0.009**	**<0.001**
LGE, %	4.1 (2.2–7.8)	0.2 (0.1–0.5)	**<0.001**	0.5 (0.3–1.5)	0.2 (0.2–0.7)	**0.019**	**<0.001**
ECV, %	27.7±4.2	24.7±3.5	**0.016**	25.8±2.9	25.1±3.0	0.404	0.059
Septal native T1, ms	1578±54	1530±46	**<0.001**	1574±49	1546±40	**0.027**	0.751
Septal T2, ms	40.9±3.8	38.2±1.9	**<0.001**	38.8±2.9	37.9±1.6	0.157	**0.021**
Septal LGE, relative % of segment	4.8 (2.4–11.9)	0.3 (0.1–0.9)	**<0.001**	1.1 (0.5–1.7)	0.3 (0.1–0.8)	**0.016**	**<0.001**
Septal ECV, %	28.6±4.8	24.8±3.9	**0.002**	25.7±3.1	24.9±3.0	0.157	**0.012**
*Cardiac DTI*							
	**MSF+** **(n=30)**^**†**^	**Controls to MSF+** **(n=28)**	***p*****-value** **MSF+*****vs.*****controls**	**MSF–** **(n=24)**^**†**^	**Controls to MSF–** **(n=24)**	***p-*****value** **MSF–*****vs.*****controls**	***p*****-value MSF +*****vs.*****MSF –**
MD, x 10^−3^ mm^2^/s	1.51 (1.47–1.54)	1.43 (1.41–1.49)	**<0.001**	1.46 (1.44–1.49)	1.44 (1.41–1.48)	0.365	**0.006**
FA	0.32 (0.30–0.36)	0.33 (0.33–0.34)	0.120	0.33 (0.32–0.35)	0.34 (0.33–0.35)	0.364	0.398
E2A, degrees	34.0±10.3	42.1±8.5	**0.003**	35.5±8.5	39.0±7.2	0.142	0.573
Septal MD, 10^−3^ mm^2^/s	1.51 (1.48–1.55)	1.44 (1.42–1.49)	**0.007**	1.44 (1.42–1.49)	1.43 (1.41–1.47)	0.403	**0.003**
Septal FA	0.30 (0.27–0.34)	0.35 (0.33–0.37)	**0.008**	0.32 (0.28–0.34)	0.36 (0.28–0.38)	0.896	0.566
Septal E2A, degrees	32.6±12.2	42.4±10.23	**0.003**	36.7±10.9	38.8±7.22	0.296	0.695
*Resting myocardial perfusion*							
	**MSF+** **(*****n***=32)*	**Controls to MSF+** **(n=28)**	***p*****-value** **MSF+*****vs.*****controls**	**MSF–** (n=26)*	**Controls to MSF–** **(n=26)**	***p-*****value** **MSF–*****vs.*****controls**	***p*****-value MSF +*****vs.*****MSF –**
Mean MBF,mL/g/min	0.64 (0.50–0.68)	0.69 (0.56–0.78)	**0.018**	0.71 (0.61–0.80)	0.70 (0.57–0.81)	0.862	**0.013**
RPPn MBF, mL/g/min	0.79 (0.67–0.90)	0.91 (0.79–1.07)	**0.016**	0.95 (0.80–1.06)	0.94 (0.80–1.23)	0.493	**0.032**
Septal MBF, mL/g/min	0.60 (0.49–0.66)	0.64 (0.56–0.75)	0.061	0.64 (0.56–0.70)	0.67 (0.55–0.76)	0.552	0.517
Septal RPPn MBF, mL/g/min	0.77 (0.58–0.89)	0.89 (0.72–1.05)	**0.016**	0.88 (0.72–1.00)	0.91 (0.74–1.11)	0.168	0.332

Values are reported as mean±SD or median (Q1–Q3). Unpaired T-test was used for normally distributed values and Wilcoxon rank-sum test for non-normally distributed variables. Septal analysis includes AHA segments 2, 3, 8, and 9. *3 DCM patients did not have myocardial perfusion data (2 MSF+,1 MSF**–**)

†16 participants did not have cDTI data (9 control and 7 DCM, comprising 4 MSF+ and 3 MSF**–**)

*CMR* cardiovascular magnetic resonances, *DTI* diffusion tensor imaging, *ECV* extracellular volume, *E2A* second eigenvector angle, *FA* fractional anisotropy, *LGE* late gadolinium enhancement, *MBF* myocardial blood flow, *MD* mean diffusivity, *MSF* midwall septal fibrosis.

### 3.3 Septal myocardial abnormalities associated with MSF

DCM patients demonstrated microstructural, perfusion, and tissue abnormalities in the septal segments, including decreased septal E2A (*p*=0.007), increased septal MD (*p*=0.003), decreased septal FA (*p*=0.002), reduced septal rMBF (*p*=0.045), increased septal native T1, T2, and ECV (all *p*≤0.01) compared to controls (Table 2).

When stratified by MSF+/−, increased septal MD was also observed (*p*=0.003), but septal FA and E2A did not differ (Table 2) in MSF+, and neither did septal perfusion metrics. MSF+ demonstrated longer septal T2 (*p*=0.021) and increased septal ECV compared with MSF− (*p*=0.012), while septal T1 did not differ (Table 3). Further subgroup comparisons are available in Table 3.

LGE+ vs. LGE− segmental analysis: within-subject mechanistic validation

To determine whether region-averaged analyses obscured localized fibrosis-related effects, within-subject comparisons of fibrotic (LGE+) and non-fibrotic (LGE−) myocardial segments were performed in MSF+ patients. As expected, LGE burden was markedly higher in LGE+ segments compared with LGE− (*p*<0.001). LGE+ segments demonstrated increased native T1 (*p*=0.029), increased ECV (*p*=0.001), and decreased rMBF and rMBF_N_ (both *p*≤0.001). No significant differences were observed in cDTI metrics or T2 between LGE+ and LGE− myocardium. (Table 4)**.**

**Table 4:** Segmental analysis within MSF+ patients comparing LGE+ to LGE– segments

**CMR metric**	**LGE+ segments**	**LGE– segments**	***p*****-value**
MD, 10^−3^ mm^2^/s	1.51 (1.44–1.58)	1.48 (1.41–1.60)	0.952
FA	0.32 (0.28–0.35)	0.34 (0.30–0.39)	0.956
E2A, degrees	33.9±16.0	33.6±16.4	0.897
Global mean rMBF, mL/g/min	0.56 (0.46–0.66)	0.66 (0.54–0.78)	**<0.001**
Global mean rMBF_N_, mL/g/min	0.77 (0.59–0.89)	0.86 (0.75–0.95)	**0.001**
Mean endocardial rMBF, mL/g/min	0.59 (0.45–0.65)	0.72 (0.63–0.82)	**<0.001**
Mean endocardial rMBF_N_, mL/g/min	0.80 (0.61–0.93)	0.95 (0.80–1.10)	**<0.001**
Mean epicardial rMBF, mL/g/min	0.53 (0.42–0.65)	0.63 (0.52–0.70)	**0.003**
Mean epicardial rMBF_N_, mL/g/min	0.75 (0.58–0.84)	0.82 (0.68–0.90)	**0.011**
Relative LGE, % of segment	13.4 (7.5–24.5)	0.0 (0.0–0.0)	**<0.001**
Native T1, ms	1578±85	1549±80	**0.0289**
Native T2, ms	40.5 (37.8–42.8)	38.8 (37.5–42.2)	0.0929
ECV,%	28.9±5.1	25.8±4.3	**0.001**

Values are reported as mean±SD or median (quartile 1–quartile 3). Linear mixed effects models were used where the fixed effect is the fibrosis status (LGE+ or LGE–) and Patient ID is included as a random effect to account for intra-patient variability. *E2A* second eigenvector angle, *ECV* extracellular volume, *FA* fractional anisotropy, *LAVi* indexed left atrial volume, *LGE* late gadolinium enhancement, *LV* left ventricular, *LVEF* left ventricular ejection fraction, *LVEDVi* left ventricular end-diastolic volume, indexed, *LVESVi* indexed left ventricular end systolic volume, *LVMassi* left ventricular mass indexed, *rMBFN* resting myocardial blood flow normalized to rate pressure product, *MD* mean diffusivity, *MWT* maximum wall thickness, *RPP* rate pressure product, *RVEF* right ventricular ejection fraction, *RVEDVi* indexed right ventricular end diastolic volume, *RVESVi* indexed right ventricular end systolic volume, *ACEi* angiotensin converting enzyme inhibitor, *ARB* angiotensin II receptor blocker, *ARNI* angiotensin receptor/nephrolysin inhibitor, *BAG3* Bcl2-associated athanogene 3, *BSA* body surface area, *DBP* diastolic blood pressure, *DCM* dilated cardiomyopathy, *DSP* desmoplakin, *MRA* mineralocorticoid receptor antagonist, *MSF* midwall septal fibrosis, *MYH7* β-myosin heavy chain 7, *NSVT* nonsustained ventricular tachycardia, *NYHA* New York Heart Association, *P/LP* pathogenic/likely pathogenic, *SBP* systolic blood pressure, *SGLT2* sodium/glucose co-transporter II inhibitor, *SVE* supraventricular ectopy, *T2DM* type 2 diabetes mellitus, *TTN* titin, *VE* ventricular ectopy, *VUS* variant of uncertain significance

### 3.4 Associations between CMR and clinical markers

#### 3.4.1 Correlation analyses

Across all participants, markers of myocardial fibrosis and interstitial expansion correlated with adverse microstructural remodeling. Native T1, ECV, and LGE mass correlated positively with MD (*r*=0.163, *p*=0.096; *r*=0.299, *p*=0.004; and *r*=0.509, *p*<0.001, respectively) and inversely with E2A (*r*=-0.239, *p*=0.014; *r*=-0.049, *p*=0.00; *r*=-0.194, *p*=0.049, respectively), with the strongest association observed between LGE mass and MD. Resting MBF correlated positively with FA (*r*=0.241; *p*=0.015) and inversely with MD (*r*=-0.309; *p*=0.002), indicating preserved microstructural integrity in better-perfused myocardium (Fig. 3**F**). Within the DCM cohort, T2 and ECV correlated positively with MD and inversely with E2A (T2 and MD: *r*=0.473, ECV and MD: *r*=0.499, both *p*<0.001; T2 and E2A (*r*=-0.367, *p*=0.006; T2 and ECV: *r*=-0.300, *p*=0.043). LVEF was strongly positively correlated with rMBF (*r* [55]: 0.405, *p*=0.002). LGE burden and ventricular ectopy burden were both inversely associated with rMBF.

Additional correlations are reported in **Supplemental Materials, Tables S4–S10**.

### 3.5 Univariate regression analyses

On univariate modeling, increased MD was associated with increasing age, greater LGE burden, increased ECV, and lower LVEF. FA showed a modest inverse association with age, while E2A was positively associated with LVEF. (**Supplemental Materials, Table S11).**

Global myocardial perfusion was associated with male sex, LGE burden, and LVEF. (exp [*β*] for male sex: 0.863, *p*<0.00; exp [*β*] for LGE: 0.977/gram, *p*<0.001; exp [*β*] for LVEF: 1.006/percent, *p*<0.00. Increased ventricular ectopy burden was also associated with decreased rMBF (exp [*β*] for VE: 0.993/percent burden, *p*<0.018). (**Supplemental Materials, Table S12).**

### 3.6 Association with ventricular ectopy burden

On univariate analysis, greater LGE percentage and decreased rMBF were associated with ventricular ectopy or NSVT. After adjustment for collinearity, decreased rMBF remained independently associated with VE/NSVT presence (OR 0.94, *p*=0.028). (**Supplemental Materials, Table S13–14).**

### 3.7 Stratification by left ventricular ejection fraction

The DCM cohort (n=61) was stratified into HFrEF (n=17), HFmrEF (n=15), and HFpEF (n=29).

Global cDTI metrics demonstrated no significant differences in MD or FA across LVEF strata. However, global E2A was significantly decreased in HFrEF compared with HFpEF patients (*p*=0.009), indicating greater impairment of sheetlet configuration with more advanced systolic dysfunction (Fig. 3**B** and **Supplementary Material Table S2**). In contrast, myocardial perfusion demonstrated a strong relationship with LVEF severity. Absolute global resting myocardial blood flow was significantly reduced in HFrEF compared with both HFmrEF and HFpEF (both *p*=0.001). No significant difference between HFmrEF and HFpEF groups was observed (Fig. 4**D** and **Supplementary Material Table S2).**

## 4 Discussion

This study combines population-level, subgroup-level, and within-subject analyses to comprehensively evaluate the role of midwall septal fibrosis in patients with DCM. The observed associations between fibrosis burden, impaired myocardial perfusion, microstructural disorganization, and ventricular arrhythmia burden support a mechanistic framework in which midwall septal fibrosis marks a transition to a structurally and functionally vulnerable myocardial substrate. Given the growing body of evidence linking the presence of MSF with major adverse arrhythmic events in DCM regardless of LVEF [33], these novel pathophysiological insights have the potential to refine patient risk stratification and inspire new drug therapies aimed at improving the myocardial microstructure and perfusion in DCM.

The increase in MD observed in our DCM cohort aligns with the current histopathological understanding of DCM [4]. MD is a measure of the freedom of water molecule diffusion; higher values indicate less restricted movement. Pathological processes that disrupt the tightly packed myocardial structure, such as interstitial fibrosis or myocyte loss, create more extracellular space and therefore allow for greater water diffusion. While other factors like edema can also elevate MD, our finding of increased global MD is in keeping with the presence of diffuse interstitial fibrosis in DCM [4], [34]. We also observed increased septal MD in MSF+ compared to MSF−, suggesting that focal fibrosis also plays a role in microstructural disruption.

Interestingly, in the exploratory within-subject LGE segmental analysis (where we compared LGE+ vs. LGE− segments within the same MSF+ patient), we found no statistically significant differences in the cDTI metrics between LGE+ and LGE− segments. However, we observed that MD was numerically higher in both LGE+ and LGE− segments (1.51 and 1.48×10^−3^ mm^2^/s, respectively), compared to the global MD value of the control cohort (1.44×10^−3^ mm^2^/s). This could suggest that in the MSF+ phenotype, the diffuse interstitial fibrosis burden in the remote myocardium (LGE−) is of similar magnitude to the microstructural abnormalities within focal fibrotic segments (LGE+). This finding is hypothesis-generating and could suggest that MD may serve as a potential marker for total fibrotic disease burden, capturing both focal and diffuse fibrosis, though it cannot differentiate between these two patterns in this cohort.

E2A is an index of mean intravoxel sheetlet angle, with lower values being abnormal and suggesting a less contracted sheetlet configuration and loss of sheetlet angularity. We consistently found reduced global and septal E2A in the DCM cohort compared to controls. However, E2A did not differ significantly between MSF+ and MSF–, indicating that disruption of sheetlet configuration may represent a more generalized DCM feature rather than a specific marker of MSF. This was supported by the findings from the within-subject LGE segmental analysis, which showed abnormally reduced E2A angles in both LGE+ and LGE– segments, suggesting a diffuse sheetlet perturbance in this subgroup. Our findings are consistent with a recent cDTI study [35] which, using a stimulated echo acquisition mode (STEAM) sequence, showed normal diastolic and reduced systolic E2A in DCM patients. While we did not assess cDTI biomarkers in diastole, our findings of reduced E2A in systole are consistent with the findings of this study [35].

We did not find significant consistent FA differences across our cohorts, aside from the septal analysis where septal FA was decreased in the DCM cohort, and in MSF+. FA is a scalar unitless value which measures restrictiveness to water diffusion. An FA value towards zero reflects less coherence of cardiomyocyte orientation, and a value towards 1 reflects increased coherence [14]. It should be noted that the histopathology of DCM is non-specific [36], [37] with several possible features known to coexist, including cardiomyocyte atrophy, nuclear pleomorphism, and interstitial fibrosis. Previous studies showed an inverse correlation between FA and histological measurements of collagen (a major component of fibrotic or scar tissue), so it is plausible that FA declines in areas of fibrosis in DCM. As we found no consistent differences in FA between our groups, this suggests that FA may be less sensitive to the pathological processes driving MSF.

To date, there have only been a few small studies using cDTI techniques in DCM. A previous study [38] that compared 9 DCM patients to controls found a trend towards increased MD, reduced FA, and reduced dynamic change in E2A in patients with DCM vs. controls. Another study found that the myocardium in pre-transplant patients with end-stage DCM had decreased FA and increased MD than control myocardium [39]; with MD being positively correlated with total collagen. Another study undertook serial cDTI imaging in patients with DCM and found that systolic E2A and sheetlet mobility remained significantly reduced in the recovered DCM compared with controls, suggesting that microstructural abnormalities persist despite normalization of LV size and LVEF [40]. In the setting of myocardial infarction, increased MD has been previously found to correspond with LGE+ segments [13], [41], [42]. While not directly comparable due to differences in methodology, overall, our data corroborates those from previous studies.

### 4.1 Perfusion and fibrosis: a regional relationship

Global rMBF was reduced in patients with DCM compared to controls; with the greatest reduction observed in MSF+ patients. Importantly, MSF– patients showed no significant perfusion differences compared with controls. In the within-subject segmental analysis, LGE+ showed decreased rMBF compared to LGE–, indicating that impairment in perfusion is spatially associated with fibrotic myocardium and closely tracks with the presence of fibrosis, lending credence to the theory that chronic myocardial hypoperfusion or microvascular ischemia is one of the key pathophysiological drivers of DCM, leading to fibrosis, adverse remodeling, and progressive myocardial functional deterioration. Furthermore, the independent association between reduced MBF and ventricular ectopy suggests that perfusion abnormalities may contribute to arrhythmogenesis beyond the presence of fibrosis alone.

Previous studies have detected such MBF abnormalities even at the earliest stages of DCM; in addition, low rMBF is a reliable major predictor of adverse cardiovascular events [43]. We hypothesize that in patients with DCM, rMBF impairment is likely to be multifactorial due to: 1) myocardial structural abnormalities, 2) the effects of increased hemodynamic load on the coronary microvascular bed, 3) structural abnormalities of the vessels and reduced capillary density, in conjunction with abnormal endothelial function [44]; and 4) coronary endothelial dysfunction, which affects MBF regulation [45]. Interestingly, a recent DCM study [46] found increased rest MBF in a heterogenous group of DCM patients when compared to controls, which at face value appears at odds with our findings and other previous myocardial perfusion DCM studies [47], [48]. However, closer inspection of the data reveals that a 16-patient DCM subgroup with MSF *did* exhibit a non-significant reduction in rest MBF when compared to controls and furthermore, fibrotic myocardial segments had decreased rest MBF, which is in keeping with our results.

### 4.2 The clinical context: linking arrhythmia, fibrosis, and perfusion abnormalities

Although an exploratory analysis only, our study showed an increase in VE/NSVT burden in the MSF+ group compared to MSF**–**. This was in spite of the MSF+ group being on more guideline-directed medical therapy (GDMT) than their MSF– counterparts (44% [15/34] of MSF+ were on 4 classes of heart failure GDMT, compared to 15% [4/27] in MSF**–,** *p*=0.02); 88% [30/34] of MSF+ were on beta-blockers compared to 74% [20/27] in MSF**–,** *p*=0.19). Proposed mechanisms include fibrosis-promoting myocyte electrotonic uncoupling to enable focal automaticity, ischemia altering cellular excitability or local stretch causing mechanoelectric feedback and activation of stretch receptors leading to triggered activity. A recent study explored mechanisms of arrhythmogenesis using computational simulation, and found that myocardial regions with both hypoxia and fibrosis generated ectopic beats, but simulations involving only one of the processes did not show ectopic beats [49]. Our study appears consistent with this: we found reduced septal rMBF in MSF+ compared to both MSF– and control groups, and increased VE burden in MSF+ compared to MSF–. In addition, our multivariable analysis demonstrated that decreased rMBF retained significant and independent association with VE even after adjusting for the burden of LGE.

Approximately 30% of DCM cases have a pathogenic or likely pathogenic (P/LP) variants [50], with titin (*TTN*) being the most frequent [51]. We observed a higher prevalence of P/LP *TTN* variants in the MSF+ cohort compared to MSF–. This is supported by previous CMR studies that have similarly reported a trend for midwall septal fibrosis in patients carrying PLP *TTN* variants [52], [53]. Larger multi-center studies are needed to confirm this potential genotype-phenotype correlation in patients with TTN variants.

In DCM histopathological studies have shown that MSF is predominantly comprised replacement fibrosis [5], which occurs after cardiomyocyte death. The precise cause of MSF is still debated, but increased septal wall stress causing direct mechanical injury to cardiomyocytes over time are likely contributors. This mechanical injury is aggravated by microvascular ischemia and activation of immune and neurohormonal axes, leading to the production of profibrotic mediators such as angiotensin II and aldosterone [5]. Similarly, mid-myocardial lesions have been observed in patients with type 2 diabetes, though mainly affecting the basal lateral and basal inferolateral myocardium instead of the septum; this is thought to be driven by altered cellular metabolism and hyperglycemia resulting in oxidative stress, microvascular angiopathy and diffuse interstitial fibrosis [54]. Although many anti-fibrotic therapies targeting cardiac fibrosis have shown promise in preclinical models, clinical translation has been disappointing [55]. There is currently no anti-fibrotic drug that has clearly demonstrated the regression of myocardial fibrosis and equivalent health improvement in clinical trials [55], [56]. Recently, autologous chimeric antigen receptor T cell-based technology has emerged as a potential therapy to treat cardiac fibrosis [57], though this is nascent.

## 5 Limitations

Limitations of our study include the loss of data due to artifact: for cDTI, 7.9% (133/1680) of segments contained artifact so were excluded. However, most of these affected segments involved the lateral wall (57%, 956/1680), sparing the septal segments which was the focus of our study. Similarly, there was a small loss of perfusion data due to artifact (3.8%, 72/1888). Although reduced resting myocardial blood flow was statistically associated with ventricular ectopy burden, the absolute ectopic burden in this cohort was low, and this relationship should therefore be interpreted cautiously. In addition, the exclusion of patients with ICDs may have biased the results towards a lower observed incidence of VE. Furthermore, the absence of a statistically significant association between LGE burden and VE presence may reflect limited statistical power or undersampling of arrhythmia burden due to the use of 24-hour ambulatory ECG monitoring rather than more extended rhythm surveillance. Extended monitoring may be required to more fully capture the relationship between myocardial fibrosis, perfusion abnormalities, and ventricular arrhythmogenesis. Additionally, the study was performed on a single 3 Tesla scanner meaning that results are therefore not immediately generalizable to the wider DCM population in a mixed healthcare setting; however, our single-magnet study design does have advantages in that all collected cDTI and multiparametric mapping data were unconfounded by site-specific hardware, software, and prototype variations. Lastly, the DCM cohort was genetically heterogenous (**Supplementary Table S1)**—further work is needed to understand gene-specific pathomechanisms of MSF.

## 6 Conclusion

Midwall septal fibrosis in DCM reflects a distinct and clinically meaningful disease phenotype, underpinned by microstructural disease and regional myocardial hypoperfusion. These abnormalities may be linked to arrhythmogenesis and may mechanistically account for the heightened risk of sudden cardiac death observed in this population. Recognition of midwall fibrosis as more than a passive marker—but rather an active pathophysiological substrate—opens the door to imaging-guided risk stratification and novel targeted preventative strategies in DCM.

## Author contributions

**Fiona TS Chan:** Writing – review & editing, Writing – original draft, Visualization, Project administration, Methodology, Investigation, Funding acquisition, Formal analysis, Data curation. **Sam Coveney:** Writing – review & editing, Data curation. **Sean L. Zheng:** Writing – review & editing, Formal analysis, Data curation. **Matthew Webber:** Writing – review & editing, Investigation. **George Joy:** Writing – review & editing, Investigation, Data curation. **Hunain Shiwani:** Writing – review & editing, Data curation. **Constantin-Cristian Topriceanu:** Writing – review & editing, Formal analysis. **Debbie Falconer:** Writing – review & editing, Data curation. **Emma Martin:** Writing – review & editing, Data curation. **Matthew Stanley:** Writing – review & editing, Data curation. **Iain Pierce:** Writing – review & editing, Supervision, Resources. **Irvin Teh:** Writing – review & editing, Supervision. **Jurgen Schneider:** Writing – review & editing, Supervision. **Christopher Nguyen:** Writing – review & editing, Software. **Alun D. Hughes:** Writing – review & editing, Supervision. **James C. Moon:** Writing – review & editing, Data curation. **Pier D. Lambiase:** Writing – review & editing, Conceptualization. **Peter Kellman:** Writing – review & editing, Software. **Erica Dall’Armellina:** Writing – review & editing, Supervision. **Gabriella Captur:** Writing – review & editing, Supervision, Project administration, Methodology, Funding acquisition, Formal analysis.

## Declaration of competing interests

The authors declare that they have no known competing financial interests or personal relationships that could have appeared to influence the work reported in this paper.
