# Supplementary material for: Microstructural disease and hypoperfusion in dilated cardiomyopathy underpin midwall septal fibrosis
Source: J Cardiovasc Magn Reson. 2026 May 29;28(2):102749. doi: 10.1016/j.jocmr.2026.102749 (PMC13332009; doi:10.1016/j.jocmr.2026.102749)
Supplement: Supplementary file 1 — Supplementary material [file mmc1.docx]

**Microstructural disease and hypoperfusion in dilated cardiomyopathy underpin midwall septal fibrosis**

Fiona TS Chan^1,2,3^, Sam Coveney^4^, Sean L Zheng^5,6^, Matthew Webber^1,2^, George Joy^2^, Hunain Shiwani^2,7^, Constantin-Cristian Topriceanu^1,2^, Debbie Falconer^1,2^, Emma Martin^1^, Matthew Stanley^3^, Iain Pierce^2,7^, Irvin Teh^4^, Jurgen Schneider^4^, Christopher Nguyen^9^, Alun D Hughes^1,2^, James C Moon^2.7^, Pier D Lambiase^2,7^, Peter Kellman^2,10^, Erica Dall’Armellina^4^*, Gabriella Captur^1,2,3^*

^*Joint last authors^

**Affiliations**

1. UCL Unit for Lifelong Health and Ageing, University College London, London, United Kingdom

2. UCL Institute of Cardiovascular Science, University College London, London, United Kingdom

3. Royal Free Hospital, Centre for Inherited Cardiac Conditions, Cardiology Department, Pond Street, Hampstead, London, United Kingdom

4. Biomedical Imaging Science Department, Leeds Institute of Cardiovascular and Metabolic Medicine, University of Leeds, Leeds, United Kingdom

5. National Heart and Lung Institute, Imperial College London, London, United Kingdom

6. Laboratory of Medical Sciences, Medical Research Council, London, United Kingdom

7. Barts Heart Centre, the Cardiovascular Magnetic Resonance Unit, London, United Kingdom

8. Department of Biomedical Engineering, King’s College London, London, United Kingdom

9. Cardiovascular Innovation Research Center; Heart, Vascular, and Thoracic Institute, Cleveland Clinic, Cleveland, Ohio, USA

10. National Institutes of Health, National Heart, Lung, and Blood Institute, Bethesda, Maryland, USA

**Supplemental Material**

| **Table S1** | List of pathogenic/likely pathogenic variants and variants of uncertain significance……………………………... | 3 |
| --- | --- | --- |
| **Table S2** | cDTI, tissue characterization, and perfusion biomarkers in the DCM cohort, stratified by LVEF………..………. | 4 |
| **Table S3** | Baseline and CMR characteristics for ‘true DCM’ versus NDLVC……………………………………………….. | 5 |
| **Table S4** | Correlation analysis for cDTI parameters in all study participants………………………………………………… | 6 |
| **Table S5** | Correlation analysis for perfusion parameters in all study participants……………………………………………. | 7 |
| **Table S6** | Correlation analysis for cDTI parameters in pooled NDLVC/DCM patients……………………………………… | 8 |
| **Table S7** | Correlation analysis for perfusion parameters in pooled NDLVC/DCM patients………………………………….. | 9 |
| **Table S8** | Correlation analysis for cDTI parameters in MSF+ only, global………………………………………….……….. | 10 |
| **Table S9** | Correlation analysis for cDTI parameters in MSF+ only, septal…………………………………………………… | 11 |
| **Table S10** | Correlation analysis for perfusion parameters in MSF+ only……………………………………………………… | 12 |
| **Table S11** | Univariate analysis for the association between clinical/CMR metrics and cDTI parameters…………………….. | 13 |
| **Table S12** | Univariate analysis for the association between clinical/CMR metrics and global rMBF………………………… | 14 |
| **Table S13** | Univariate glm regression for predictors of VE/NSVT presence……………………………………….................. | 15 |
| **Table S14** | Multivariable glm regression for significant predictors of VE/NSVT presence…………………………………… | 16 |

**Table S1.** List of pathogenic/likely pathogenic variants and variants of uncertain significance identified in DCM cohort (all other study patients were gene elusive).

| **Classification** | **Gene** | **Transcript** | **DNA variant** | **Protein variant** | **Genomic location** | **Zygosity** |
| --- | --- | --- | --- | --- | --- | --- |
| LP | MYH7 | NM_000257.2 | c.452C>T | p.(Pro151Leu) | Chr14:23432689 | Heterozygous |
| LP | TTN | NM_001267550.1 | c.43544dup | p.(Phe14516llefsTer8) | Chr2: 178632349-178632350 | Heterozygous |
| LP | TTN | NM_001267550.2 | c.53393del | p.(Gly17798AlafsTer18) | Chr2: 178807209 | Heterozygous |
| LP | TTN | NM_001267550.1 | c.91356dup | p.(Lys30453Ter) | Chr2: 178551174-178551175 | Heterozygous |
| LP | TTN | NM_001267550.1 | c.94452T>A | p.(Tyr31484Ter) | Chr2: 178547073 | Heterozygous |
| LP | TTN | NM_001267550.1 | c.97129_97130dup | p.(Leu32379HisfsTer4) | Chr2: 178542723-178542724 | Heterozygous |
| LP | TTN | NM_001267550.1 | c.48145_48224del | p.(Asp16049Leufs*5) | Chr2: 178616567-178616744 | Heterozygous |
| LP | TTN | NM_001267550.1 | c.96092_96095del | p.(Ser32031CysfsTer3) | Chr2: 178544049-178544052 | Heterozygous |
| LP | BAG3 | NM_004281.4 | c.1077del | p.(Glu360ArgfsTer3) | Chr10: 119676631 | Heterozygous |
| LP | FLNC | NM_001458 | c.4969C>T | p.(Arg1657Ter) | Chr7: 128849348 | Heterozygous |
| VUS | TMEM43 | NM_024334.2 | c.322G>T | p.(Val108Phe) | Chr3: 14131604 | Heterozygous |
| VUS | MYH7 | NM_000257.2 | c.745del | p.(Arg249GlufsTer15) | Chr14:23431469 | Heterozygous |
| VUS | MYH7 | NM_000257.2 | c.1700G>A | p.(Arg567His) | Chr14: 23427773 | Heterozygous |
| VUS | MYH7 | NM_000257.2 | c.2423+1G>A |  | Chr14: 23425281 | Heterozygous |
| VUS | TTN | NM_001267550.1 | c.3109G>T | p.(Glu1037Ter) | Chr2: 178782594 | Heterozygous |
| VUS | TTN | NM_001267550.1 | c.12907del | p.(lle4303PhefsTer2) | Chr2: 178740326 | Heterozygous |
| VUS | FLNC | NM_001458 | c.734T>A | p.(Val245Glu) | Chr7: 128837432 | Heterozygous |

*LP, likely pathogenic; VUS, variant of uncertain significance.*

**Table S2.** cDTI, tissue characterization, and perfusion biomarkers in the DCM cohort, stratified by LVEF.

| **CMR metric** | **HFrEF**  **LVEF ≤40%**  **(n=17)** | **HFmrEF**  **LVEF 41-49%**  **(n=15)** | **HFpEF LVEF ≥50%**  **(n=29)** | ***p*-value**  **HFrEF *vs.* HFmrEF** | ***p-*value**  **HFrEF *vs.***  **HFpEF** | ***p*-value**  **HFmrEF *vs.* HFpEF** |
| --- | --- | --- | --- | --- | --- | --- |
| MD, x 10^-3^ mm^2^/s | 1.51 (1.49-1.55) | 1.49 (1.45-1.52) | 1.47 (1.45-1.52) | 0.410 | 0.223 | 0.985 |
| FA | 0.32 (0.31-0.37) | 0.33 (0.32-0.36) | 0.32 (0.30-0.33) | 0.990 | 0.431 | 0.379 |
| E2A, degrees | 28.5±9.8 | 34.9±7.2 | 37.8±8.5 | 0.188 | **0.009** | 0.603 |
| Mean MBF,ml/g/min | 0.49 (0.41-0.66) | 0.71 (0.64-0.75) | 0.66 (0.61- 0.75) | **0.001** | **0.001** | 0.883 |
| RPPn mean MBF, ml/g/min | 0.74 (0.51-0.77) | 0.89 (0.81-1.00) | 0.90 (0.79-1.05) | **0.010** | **<0.001** | 0.681 |
| Native T1, ms | 1577±66 | 1560±38 | 1527**±**43 | 0.611 | **0.005** | 0.100 |
| T2, ms | 39.2 (37.9-41.5) | 38.6 (37.8-39.8) | 38.4 (37.0-39.7) | **0.886** | 0.644 | 0.938 |
| ECV, % | 28.6±4.6 | 27.4±3.3 | 25.6**±**3.2 | 0.654 | **0.045** | 0.343 |

Abbreviations as in main manuscript Tables.

**Table S3.** CMR characteristics for classical DCM versus DCM with LV remodeling. ‘Classical’ DCM was defined and LVEF ≤50% and LVEDVi ≥110ml/m2 for men, or ≥95ml/m2 for women. DCM with LV remodeling was defined as LVEF>50% or LVEDVi <110ml/m^2^ for men or <95ml/m^2^ for women.

| **Characteristic** | **Classical DCM**  **(n=21)** | **DCM with LV remodeling (n=40)** | ***p*-value** |
| --- | --- | --- | --- |
| *Structure and function* | | | |
| MWT | 10.8 (9.9-12.1) | 11.1 (9.8-12.7) | 0.755 |
| LVMassi (g/m^2^) | 74 (67-86) | 59 (53-68) | **<0.001** |
| LVEDVi (ml/m^2^) | 123 (114-139) | 96 (81-101) | **<0.001** |
| LVESVi (ml/m^2^) | 84 (68-100) | 41 (35-51) | **<0.001** |
| LVEF, % | 32 (27-41) | 53 (48-58) | **<0.001** |
| RVEDVi (ml/m^2^) | 105±33 | 91±19 | **0.065** |
| RVESVi (ml/m^2^) | 52 (43-86) | 46 (36-53) | 0.032 |
| RVEF (%) | 42±15 | 51±9 | 0.024 |
| *Tissue characterization* | | | |
| Native global T1^§^, ms | 1571 (1550-1596) | 1532 (1510-1554) | **0.004** |
| Global T2^§^, ms | 39.0 (37.5-40.9) | 38.5 (37.5-39.7) | 0.603 |
| LGE mass, g | 2.37 (0.69-5.45) | 0.57 (0.14-1.42) | **0.002** |
| LGE volume, % | 4.2 (1.7-7.7) | 1.2 (0.4-3.1) | **0.008** |
| ECV, % | 28.2±4.3 | 26.1±3.3 | 0.079 |
| *First pass perfusion* | | | |
| Global mean rMBF, ml/g/min | 0.58±0.14 | 0.68±0.13 | **0.024** |
| Global mean rMBF_N_, ml/g/min | 0.76±0.15 | 0.90±0.21 | **0.006** |
| *Cardiac DTI* | | | |
| Global MD, x10^-3^mm^2^/s | 1.51 (1.47-1.54) | 1.47 (1.44-1.52] | 0.062 |
| Global FA | 0.33±0.03 | 0.32 ±0.03 | 0.226 |
| Global E2A, degrees | 29.5±7.9 | 37.5 ±9.1 | **0.002** |

Abbreviations as in main manuscript Tables.**Table S4.** Correlation analysis for cDTI parameters in all study participants.

|  | **MD** | | | **FA** | | | **E2A** | | |
| --- | --- | --- | --- | --- | --- | --- | --- | --- | --- |
|  | Correlation coefficient (ρ) | *p*-value | DF | Correlation coefficient (ρ) | *p*-value | DF | Correlation coefficient (ρ) | *p*-value | DF |
| LVEDVi | 0.239 | **0.014** | 103 | 0.111 | 0.260 | 103 | -0.377 | **<0.001** | 103 |
| LVEF | -0.417 | **<0.001** | 103 | 0.042 | 0.672 | 103 | 0.439 | **<0.001** | 103 |
| T1 | 0.163 | 0.096 | 103 | 0.151 | 0.123 | 103 | -0.239 | **0.014** | 103 |
| T2 | 0.202 | **0.040** | 102 | 0.14 | 0.156 | 102 | -0.422 | **<0.001** | 102 |
| ECV | 0.299 | **0.004** | 89 | -0.053 | 0.618 | 89 | -0.426 | **<0.001** | 89 |
| LGE mass | 0.509 | **<0.001** | 101 | -0.233 | **0.018** | 101 | -0.194 | **0.049** | 101 |
| rMBF | -0.148 | 0.139 | 99 | 0.076 | 0.452 | 99 | 0.083 | 0.408 | 99 |
| rMBF_N_ | -0.309 | **0.002** | 99 | 0.241 | **0.015** | 99 | -0.093 | 0.356 | 99 |

*DF, degrees of freedom.* Other abbreviations as in main manuscript Tables.

**Table S5.** Correlation analysis for perfusion parameters in all study participants.

|  | **rMBF** | | | **rMBF_N_** | | |
| --- | --- | --- | --- | --- | --- | --- |
|  | Correlation coefficient (ρ) | *p*-value | DF | Correlation coefficient (ρ) | *p*-value | DF |
| LVEDVi | -0.292 | **0.001** | 114 | -0.082 | 0.380 | 114 |
| LVEF | 0.278 | **0.002** | 114 | 0.279 | **0.002** | 114 |
| T1 | 0.057 | 0.545 | 114 | 0.038 | 0.687 | 114 |
| T2 | 0.001 | 0.995 | 113 | 0.107 | 0.257 | 113 |
| ECV | -0.06 | 0.552 | 98 | 0.043 | 0.671 | 98 |
| LGE mass | -0.365 | **<0.001** | 112 | -0.406 | **<0.001** | 112 |
| MD | -0.148 | 0.139 | 99 | -0.309 | **0.002** | 99 |
| FA | 0.076 | 0.452 | 99 | 0.241 | **0.015** | 99 |
| E2A | 0.083 | 0.408 | 99 | -0.093 | 0.356 | 99 |

Abbreviations as in **Table S3** or main manuscript Tables.

**Table S6.** Correlation analysis for cDTI parameters in DCM patients.

|  | **MD** | | | **FA** | | | **E2A** | | |
| --- | --- | --- | --- | --- | --- | --- | --- | --- | --- |
|  | Correlation coefficient (ρ) | *p*-value | DF | Correlation coefficient (ρ) | *p*-value | DF | Correlation coefficient (ρ) | *p*-value | DF |
| LVEDVi | 0.212 | 0.124 | 52 | 0.183 | 0.186 | 52 | -0.405 | **0.002** | 52 |
| LVEF | -0.28 | **0.040** | 52 | -0.177 | 0.199 | 52 | 0.420 | **0.002** | 52 |
| T1 | 0.155 | 0.264 | 52 | 0.187 | 0.175 | 52 | -0.215 | 0.119 | 52 |
| T2 | 0.473 | **<0.001** | 52 | 0.037 | 0.792 | 52 | -0.367 | **0.006** | 52 |
| ECV | 0.499 | **<0.001** | 44 | -0.221 | 0.139 | 44 | -0.300 | **0.043** | 44 |
| LGE mass | 0.490 | **<0.001** | 50 | -0.257 | **0.066** | 50 | -0.186 | 0.187 | 50 |
| rMBF | -0.142 | 0.321 | 49 | 0.038 | 0.789 | 49 | 0.050 | 0.729 | 49 |
| rMBF_N_ | -0.126 | 0.379 | 49 | 0.063 | 0.660 | 49 | -0.068 | 0.634 | 49 |
| VA burden | 0.278 | 0.087 | 38 | -0.052 | 0.754 | 52 | -0.211 | 0.198 | 38 |

Abbreviations as in **Table S3** or main manuscript Tables.

**Table S7.** Correlation analysis for perfusion parameters in DCM patients.

|  | **Global rMBF** | | | **Global rMBF_N_** | | | **Septal rMBF** | | | **Septal rMBF_N_** | | |
| --- | --- | --- | --- | --- | --- | --- | --- | --- | --- | --- | --- | --- |
|  | Correlation coefficient (ρ) | *p*-value | DF | Correlation coefficient (ρ) | *p*-value | DF | Correlation coefficient (ρ) | *p*-value | DF | Correlation coefficient (ρ) | *p*-value | DF |
| LVEDVi | -0.332 | 0.012 | 55 | -0.186 | 0.165 | 55 | -0.311 | **0.017** | 55 | -0.165 | 0.225 | 55 |
| LVEF | 0.405 | **0.002** | 55 | 0.468 | **<0.001** | 55 | 0.371 | **0.004** | 55 | 0.387 | **0.003** | 55 |
| T1 | 0.009 | 0.949 | 55 | 0.054 | 0.689 | 55 | 0.036 | 0.789 | 55 | 0.035 | 0.795 | 55 |
| T2 | -0.038 | 0.779 | 55 | 0.013 | 0.921 | 55 | 0.041 | 0.760 | 55 | 0.078 | 0.566 | 55 |
| ECV, % | -0.17 | 0.243 | 47 | 0.013 | 0.927 | 47 | 0.011 | 0.942 | 47 | 0.136 | 0.355 | 47 |
| LGE mass | -0.46 | **<0.001** | 53 | -0.367 | **0.006** | 53 | -0.364 | **0.006** | 53 | -0.292 | **0.032** | 53 |
| MD | -0.142 | 0.321 | 49 | -0.126 | 0.379 | 49 | -0.047 | 0.740 | 49 | -0.113 | 0.435 | 49 |
| FA | 0.038 | 0.789 | 49 | 0.063 | 0.660 | 49 | 0.016 | 0.913 | 49 | 0.092 | 0.524 | 49 |
| E2A | 0.050 | 0.729 | 49 | -0.068 | 0.634 | 49 | 0.111 | 0.433 | 49 | -0.034 | 0.814 | 49 |
| VA burden | -0.431 | **0.005** | 39 | -0.363 | **0.022** | 39 | -0.374 | **0.015** | 39 | -0.299 | 0.058 | 39 |

Abbreviations as in **Table S3** or main manuscript Tables.

**Table S8.** Correlation analysis for cDTI parameters in MSF+ only, global.

|  | **Global MD** | | | **Global FA** | | | **Global E2A** | |  |
| --- | --- | --- | --- | --- | --- | --- | --- | --- | --- |
|  | Correlation coefficient (ρ) | *p*-value | DF | Correlation coefficient (ρ) | *p*-value | DF | Correlation coefficient (ρ) | *p*-value | DF |
| LVEDVi | -0.038 | 0.844 | 28 | 0.408 | **0.025** | 28 | -0.520 | **0.003** | 28 |
| LVEF | -0.226 | 0.229 | 28 | -0.431 | **0.018** | 28 | 0.545 | **0.002** | 28 |
| T1 | -0.004 | 0.983 | 28 | 0.270 | 0.149 | 28 | -0.302 | 0.105 | 28 |
| T2 | 0.59 | **0.001** | 28 | -0.011 | 0.955 | 28 | -0.393 | **0.032** | 28 |
| ECV | 0.655 | **<0.001** | 23 | -0.188 | 0.367 | 23 | -0.279 | 0.177 | 23 |
| LGE (mass) | 0.371 | 0.052 | 26 | -0.168 | 0.392 | 26 | -0.271 | 0.163 | 26 |
| rMBF | 0.172 | 0.382 | 26 | -0.107 | 0.588 | 26 | 0.162 | 0.411 | 26 |
| rMBF_N_ | 0.086 | 0.662 | 26 | 0.067 | 0.735 | 26 | -0.085 | 0.668 | 26 |
| VA burden | 0.141 | 0.482 | 25 | 0.049 | 0.808 | 25 | -0.198 | 0.322 | 25 |

Abbreviations as in **Table S3** or main manuscript Tables.

**Table S9.** Correlation analysis for cDTI parameters in MSF+ only, septal.

|  | **Septal MD** | | | **Septal FA** | | | **Septal E2A** | | |
| --- | --- | --- | --- | --- | --- | --- | --- | --- | --- |
|  | Correlation coefficient (ρ) | *p*-value | DF | Correlation coefficient (ρ) | *p*-value | DF | Correlation coefficient (ρ) | *p*-value | DF |
| LVEDVi | 0.172 | 0.364 | 28 | 0.333 | 0.072 | 28 | -0.477 | **0.008** | 28 |
| LVEF | -0.249 | 0.184 | 28 | -0.403 | **0.027** | 28 | 0.636 | **<0.001** | 28 |
| T1 | 0.285 | 0.127 | 28 | 0.276 | 0.140 | 28 | -0.273 | 0.144 | 28 |
| T2 | 0.740 | **<0.001** | 28 | -0.008 | 0.967 | 28 | -0.372 | **0.044** | 28 |
| ECV | 0.682 | **<0.001** | 23 | -0.166 | 0.428 | 23 | -0.278 | 0.178 | 23 |
| LGE mass | 0.378 | **0.048** | 26 | -0.162 | 0.412 | 26 | -0.265 | 0.172 | 26 |
| rMBF | 0.003 | 0.988 | 26 | -0.162 | 0.411 | 26 | 0.386 | **0.043** | 26 |
| rMBF_N_ | 0.144 | 0.462 | 26 | 0.038 | 0.846 | 26 | 0.18 | 0.348 | 26 |
| VA burden | 0.224 | 0.262 | 25 | -0.02 | 0.922 | 25 | -0.206 | 0.302 | 25 |

Abbreviations as in **Table S3** or main manuscript Tables.

**Table S10.** Correlation analysis for perfusion parameters in MSF+ only.

| **Variables** | **Global rMBF** | | | **Global rMBF_N_** | | | **Septal rMBF** | | | **Septal rMBF_N_** | | |
| --- | --- | --- | --- | --- | --- | --- | --- | --- | --- | --- | --- | --- |
|  | Correlation coefficient (ρ) | *p-*value | DF | Correlation coefficient (ρ) | *p*-value | DF | Correlation coefficient (ρ) | *p*-value | DF | Correlation coefficient (ρ) | *p*-value | DF |
| LVEDVi | -0.231 | 0.211 | 29 | 0.027 | 0.884 | 29 | -0.090 | 0.623 | 29 | 0.126 | 0.494 | 29 |
| LVEF | 0.545 | **0.002** | 29 | 0.435 | 0.014 | 29 | 0.419 | 0.017 | 29 | 0.309 | 0.085 | 29 |
| T1 | -0.005 | 0.978 | 29 | 0.130 | 0.487 | 29 | 0.009 | 0.962 | 29 | 0.110 | 0.548 | 29 |
| T2 | -0.077 | 0.679 | 29 | 0.146 | 0.432 | 29 | 0.101 | 0.580 | 29 | 0.243 | 0.179 | 29 |
| ECV | -0.284 | 0.160 | 24 | -0.066 | 0.748 | 24 | 0.049 | 0.809 | 24 | 0.085 | 0.671 | 24 |
| LGE | -0.311 | 0.101 | 27 | -0.199 | 0.299 | 27 | -0.240 | 0.200 | 27 | -0.117 | 0.537 | 27 |
| MD | 0.172 | 0.382 | 26 | 0.086 | 0.662 | 26 | 0.260 | 0.173 | 26 | 0.143 | 0.460 | 26 |
| FA | -0.107 | 0.588 | 26 | 0.067 | 0.735 | 26 | -0.082 | 0.673 | 26 | 0.030 | 0.878 | 26 |
| E2A | 0.162 | 0.411 | 26 | -0.085 | 0.668 | 26 | 0.096 | 0.622 | 26 | -0.020 | 0.918 | 26 |
| VA burden | -0.300 | 0.129 | 25 | -0.143 | 0.476 | 25 | -0.189 | 0.336 | 25 | -0.125 | 0.526 | 25 |

Abbreviations as in **Table S3** or main manuscript Tables.

**Table S11.** Univariate analysis for the association between clinical/CMR metrics and cDTI parameters.

| **Variables** | **MD** | | | **FA** | | | **E2A** | | |
| --- | --- | --- | --- | --- | --- | --- | --- | --- | --- |
|  | Exp(β] | *p-*value | 95% CI | Exp(β] | *p-*value | 95% CI | Exp(β] | *p-*value | 95% CI |
| **Age** | 1.002 | **<0.001** | 1.001,  1.003 | 0.999 | **0.003** | 0.999 1.000 | 1.166 | 0.0872 | 9.811 x10^-1^ , 1.387 |
| **Sex** | 0.986 | 0.468 | 0.950, 1.024 | 0.994 | 0.494 | 0.977 to 1.011 | 5.44 | 0.547 | 2.296 x10^-2^ 1.287 x10^3^ |
| **LGE mass** | 1.006 | **0.025** | 1.001,  1.011 | 0.999 | 0.283 | 0.996 to 1.001 | 9.159255 x10^-1^ | 0.817 | 4.367 x10^-1^ to 1.921 |
| **LVEF** | 0.998 | **0.005** | 0.997 to 0.999 | 0.999 | 0.108 | 0.999, 1.000 | 1.362 | **<0.001** | 1.153, 1.608 |
| **T1** | 1.000 | **0.023** | 1.000, 1.001 | 1.000 | 0.070 | 0.999, 1.000 | 9.503 x10^-1^ | **0.042** | 9.058 x10^-1^, 9.969 x10^-1^ |
| **ECV** | 1.011 | **<0.001** | 1.006, 1.015 | 0.998 | 0.139 | 0.996, 1.000 | 4.680 x10^-1^ | **0.043** | 2.301 x10^-1^, 9.552x10^-1^ |
| **rMBF** | 0.947 | 0.321 | 0.852, 1.053 | 1.008 | 0.789 | 0.954, 1.064 | 2.273 x10^1^ | 0.729 | 5.401 x10^-7^, 9.569 x10^8^ |
| **rMBF_N_** | 0.964 | 0.379 | 0.890, 1.045 | 1.009 | 0.660 | 0.968, 1.052 | 3.864 x10^-2^ | 0.634 | 6.382x10^-8^, 2.339x10^4^ |
| **VA burden** | 1.000 | 0.746 | 0.998, 1.003 | 1.000 | 0.634 | 0.999, 1.002 | 7.206 x10^-1^ | 0.114 | 4.848 x10^-1^, 1.071 |

*CI, confidence interval.* Other abbreviations as in main manuscript Tables. **Table S12.** Univariate analysis for the association between clinical/CMR metrics and global rMBF.

| **Variables** | **Global rMBF** | | |
| --- | --- | --- | --- |
|  | **Exp[*β*]** | ***p-*value** | **95% CI** |
| **Age** | 0.999 | 0.477 | 0.996, 1.002 |
| **Sex** | 0.863 | **<0.001** | 0.803, 0.929 |
| **LGE mass** | 0.977 | **<0.001** | 0.968, 0.985 |
| **LVEF** | 1.006 | **<0.001** | 1.003, 1.008 |
| **T1** | 1.000 | 0.949 | 0.999, 1.001 |
| **ECV** | 0.993 | 0.243 | 0.981, 1.005 |
| **MD** | 0.690 | 0.322 | 0.334, 1.427 |
| **FA** | 1.216 | 0.789 | 0.291, 5.073 |
| **E2A** | 1.001 | 0.729 | 0.996, 1.005 |
| **VA burden** | 0.993 | **0.018** | 0.987, 0.999 |

Abbreviations as in **Table S11** or main manuscript Tables.

**Table S13.** Univariate glm regression results for clinical and CMR predictors of VE/NSVT presence.

| **Variables** | **VA Presence** | | |
| --- | --- | --- | --- |
|  | **Exp(β]** | **95% CI** | ***p-*value** |
| **Age** | 1.02 | 0.98, 1.07 | 0.346 |
| **Sex** | 1.32 | 0.32, 5.36 | 0.696 |
| **LVEF** | 0.97 | 0.91, 1.01 | 0.087 |
| **T1** | 1.00 | 0.99, 1.02 | 0.816 |
| **T2** | 1.06 | 0.90, 1.28 | 0.828 |
| **ECV** | 1.03 | 0.88, 1.23 | 0.705 |
| **LGE %** | 1.33 | 1.06, 1.82 | **0.034** |
| **MSF+** | 2.81 | 0.76, 11.1 | 0.125 |
| **MD** | 1.15 | 1.00, 1.35 | 0.075 |
| **E2A** | 0.96 | 0.89, 1.04 | 0.323 |
| **FA** | 0.92 | 0.73, 1.14 | 0.465 |
| **rMBF** | 0.96 | 0.92, 0.99 | **0.032** |

Abbreviations as in **Table S11** or main manuscript Tables. MSF+ is a binary variable (presence/absence).

**Table S14.** Multivariable glm regression for significant predictors of VE/NSVT presence.

| **Variables** | **VA Presence** | | |
| --- | --- | --- | --- |
|  | **Exp[β]** | **95% CI** | ***p-*value** |
| rMBF, ml/g/min | 0.94 | 0.87 , 0.99 | **0.028** |
| LGE, % | 1.23 | 0.93, 1.71 | 0.178 |

Abbreviations as in **Table S11** or main manuscript Tables.
